# The fractured lens: a controversial revision of the International Classification of Primary Care

**DOI:** 10.3389/fmed.2023.1230987

**Published:** 2024-01-11

**Authors:** Jean K. Soler, Nicola Buono, Elena Cardillo, Thomas Frese, Shlomo Vinker, Mehmet Ungan

**Affiliations:** ^1^The Family Practice, Attard, Malta; ^2^Department of General Practice, ICPC Club Italia, Caserta, Italy; ^3^Institute of Informatics and Telematics, National Research Council, Rende, Italy; ^4^Institute of General Practice and Family Medicine, Medical Faculty, Martin-Luther-University Halle-Wittenberg, Halle, Germany; ^5^Department of Family Medicine, Sackler Faculty of Medicine, Tel Aviv University, Tel Aviv, Israel; ^6^Leumit Health Services, Tel Aviv, Israel; ^7^Department of Family Medicine, Ankara University School of Medicine, Ankara, Türkiye

**Keywords:** International Classification of Primary Care, family medicine, general practice, primary care, episode of care, reason for encounter

## Abstract

**Background:**

The International Classification of Primary Care (ICPC) has represented the international standard reduction for measuring the content of primary care for over 30 years. In the process of its third revision, its authors, the Wonca International Classification Committee (WICC), delegated a major part of the technical work to a purposely formed Consortium. However, in the process of such revision, standard classification principles and rules have been inconsistently applied with the result that ICPC-3 has been published with major errors and an inconsistent structure.

**Objectives:**

To formally describe and critically appraise the revision process of ICPC-3.

**Methods:**

The formal review of ICPC-3 performed by an expert group within WICC and commissioned by the Executive Council of Wonca Europe is presented in abridged form.

**Results:**

ICPC-3 as currently presented introduces major departures from formal classification principles and rules, besides other major errors and inconsistencies, all of which are listed and described.

**Conclusion:**

Major changes in ICPC-3 defy categorisation and conceptualisation standards. ICPC-3 now represents an untested departure from international standard presentations, without a formal academic base. The direct inclusion of measures of functioning in a classification of reasons for encounter and health problems fails to address the dichotomy of these domains, the boundaries of and relationships between which are not satisfactorily resolved by the system. Analysis of ICPC-3 data will require the development and implementation of alternative, as yet undefined, models of the relationships between disease and health. By including different domains without resolving ambiguity, and by splitting function from other body systems, ICPC-3 becomes an internally fractured instrument.

## Introduction to ICPC and the revision process

In 1987, International Classification of Primary Care (ICPC) was published as a tool to order the domain of family practice, using the concept of the *Episode of Care* (EoC)^1^ to capture the context of time in longitudinal care (1). It was developed formally as a theoretical classification based on explicit principles, followed the standard rules for creating distinct and defined categories (*Classes*, defined by *Rubrics*),^2^ and emerged from international empirical studies of the content of day-to-day family practice. It allowed for the documentation of both the patient’s *Reason for Encounter* (RfE)^3^ and the doctor’s diagnostic label (the title of the EoC, or *episode title*), together with *Process* of care, or intervention, elements. ICPC’s structure and content were defined by its characteristics (Table 1) (2). The importance of such defining characteristics, and specifically the EoC structure and the frequency of observations, has been previously described (2).

**Table 1:** The characteristics of ICPC.

Its purpose is to order the domain of family practice in the format of episodes of care
It provides a single terminology for the patient’s reason for encounter and the family doctor’s diagnosis, thus representing both sides of the same coin
It captures the changes (transitions) in the content of episodes of care over time
It follows strict taxonomic rules, so its classes are mutually exclusive
It offers - if possible – one class for common (occurring ≥1 per 1,000 patient years) reasons for encounter and diagnoses. Less common classes are included in ‘ragbags’^*^
Its biaxial structure (chapters for body systems/problem areas, and components identical throughout all chapters) results in 3-digit mnemonic, alphanumeric codes
Its reliability and validity are supported by its coding rules and a growing comparative international data base
In the coding process, localisation takes precedence over aetiology
Symptom diagnoses take precedence over disease diagnoses that are uncertain (i.e., do not fulfil the inclusion criteria)
It does not cater for mind–body metaphors: ‘psychosomatic’ and ‘somatoform’ disorders are not included.

^*^A residual class which includes symptoms or diseases with low prevalence, not elsewhere classified.

ICPC was published by the World Organisation of Family Doctors (Wonca) and maintained by the Wonca International Classification Committee (WICC).^4^ It has been translated in 22 languages, accepted by the World Health Organization (WHO) as a member of the Family of International Classifications (WHO/FIC), used widely for the routine collection of EoC data (e.g., in The Netherlands, Japan, Poland, Malta, Serbia) and also in encounter-based studies (e.g., in Australia, Norway, Denmark, The Netherlands), and is now supported by large empirical databases (3, 4). ICPC is therefore an international standard presentation (5).

The central role of WICC is to develop and maintain classifications and coding tools for primary care within Wonca. Through its agency and that of its members, ICPC was duly revised in 1998 as ICPC-2 and in 2005 as ICPC-2-Revised (6, 7). In both cases, its core structure and characteristics were carefully preserved, with Classes being added or removed on the basis of observed frequency. Since that time, there has been a perceived need within WICC to improve ICPC by adding space for new Classes, moving specific Classes from one *Chapter*^5^ or *Component* (see footnote 5) to another to improve consistency (see footnote 5), updating class definitions and adding more specific Classes for prevention and health promotion. There was also agreement to re-organise the social problems Chapter, and to create and link tools for measuring functional status and individual patient characteristics. Such changes were to be implemented in the third revision of ICPC.

During the annual WICC meetings between 2009 (Brazil) and 2018 (Ukraine), decisions taken to move in such a direction included changing the Rubric tag from three to four characters to enable more Classes, merging Chapters X and Y (male and female genital), and accepting new Classes with a frequency of 0.5 per thousand patient years of observation (as against 1.0^2^ previously). The revision of ICPC Classes was allocated by Chapter to working groups, together comprising most WICC members. However, little work was performed between actual WICC meetings, with the exception of annual small group meetings to update ICPC-2, its mappings to International Classification of Disease, version 10 (ICD-10) (8), and the included process codes, which also accomplished some exploratory work on the future major revision.

### ICPC-3 revision

At the WICC meeting in 2016 (Finland), WICC members agreed on the formation of an independent Consortium (9) to perform the ICPC-3 revision, with financial support from Wonca and Wonca member organisations. It was formally agreed that ICPC-3 would be owned by Wonca, authored by WICC, and that the actual revision work was to be performed by technicians employed within the new entity. The WICC Chapter groups reviewed each ICPC-2 Rubric, referring to an international database of empirical primary care data collected with ICPC-1, ICPC-2, ICPC-2-Plus (Australian extension) and ICD-10, compiled by Australian WICC members using databases provided by other WICC members. The Chapter group recommendations were to be edited and harmonised by a specific Task Group formed within WICC (of which the authors were members), also tasked with issues common to more than one Chapter. A Process Group was formed specifically to review the Process rubrics (Components 2 to 6). The Chapter groups did complete their work, with few exceptions, and such was subsequently reviewed by the WICC Task Group, with specific recommendations being documented, harmonised to core rules and principles and forwarded to the ICPC-3 Consortium for implementation. WICC, as the author of ICPC-3, was to subsequently review and approve of any and all changes to the Classification, as well as the final product as a whole.^6^

Conversely, the Consortium formed its own Core Group and “Taskforce,” and worked mostly independently from WICC. In the period between 2017 and 2019, the Consortium produced, and shared with WICC, a series of reports which were to define its work process and timeline, and also created an online ICPC-3 browser which incorporated all the changes being implemented (9). Unfortunately, the browser did not allow commenting, and there was no log of changes or versions. Additionally, there was no formal request from the Consortium to review, or provide feedback on, any of its decisions, except in very general terms. A number of decisions made independently from WICC, included changing the ICPC-3 coding structure, splitting Chapters, additions and deletions, inclusions of measures of functioning within the core (rather than as an extension), and to otherwise reverse, modify or ignore many of the specific recommendations made by the Chapter and Process groups, and even formal decisions made by WICC and its ICPC-3 Task Group.^7^

Consequently, the WICC Task Group reacted and sent two formal reports to the Consortium (Appendices 1, 2). The first, in March 2020, specified the principles and rules of classifications in general, and ICPC in particular, which had guided the Task Group’s work, and made specific proposals for appropriate changes in ICPC-3 to correctly address the perceived needs for revision. The second critically reviewed the ICPC-3 revision as presented in April 2020, and identified significant departures from the aforementioned principles and rules. This second report was formally commissioned for this specific purpose by the Council of Wonca Europe. Again, each major change in ICPC-3 was reviewed, with proposed modifications to align such with formal classification rules and historical ICPC principles. Neither report resulted in any major changes to the revision or its process. Independently, individual WICC members and Consortium members were also openly critical of many of the changes made, especially the major departures from historical norms.^8^

During the 2020 (Germany) WICC meeting, the Consortium requested the formal approval of ICPC-3. WICC members instead voted that ICPC-3 development should continue, since many expressed doubts regarding numerous major issues. Nevertheless, ICPC-3 was subsequently “field tested” without substantial modification. Wonca members were invited to freely use a “coding fun” application within the ICPC-3 browsing tool. Volunteers were presented with a randomly chosen text definition derived from a randomly selected ICPC-3 Rubric, asked to search for a matching Rubric, and to finally give their opinion whether the definition accurately represented the concept (9). The results of the test were neither published nor reviewed by WICC. ICPC-3 was subsequently published in December 2020, without testing in actual practice or any further peer review. At the time of publication there was a lack of any formal guidance on the new analysis protocols for ICPC-3 data, considering such major changes to the structure, Chapters and Components, and Classes.

## Critical review of ICPC-3

The following summarises the WICC Task Group’s review of ICPC-3, commissioned by Wonca Europe.

### Chapters and structure

In a classification, categories should be mutually exclusive. Such a rule applies to Classes in ICPC, and also to Chapters, since these latter are distinct hierarchical nodes which also define membership of a unique category (10). Such has apparently not been consistently applied with ICPC-3. Chapter A1 is the category for “Visits for general examination, routine examination, family planning, prevention, and other visits,” as a RfE (a request for an intervention) or an episode title. The exceptional two-digit Chapter alphanumeric code deviates from both ICPC-1/-2 and ICPC-3 conventions (as approved by WICC), and is not explicitly justified. The Chapter description defines its Classes as appropriate for coding when “…[there is] no apparent health problem involved.” Classes therein are thus split off from all other body system Chapters. Conceptually, this split of “no disease” preventive activity from the multi-system (and residual) Chapter A, together with split of family planning from Chapters W and Y (where they resided in ICPC-1 and ICPC-2), requires a re-definition of these Chapters since they no longer contain all the Classes which classically defined their content in ICPC-1 and -2.

The “no disease” Chapter A1 also creates another conflict with the Functioning Chapter II, which also contains concepts which are either not related to a specific disease, or define the normal state. Paradoxically, Chapter A1 contains residual Classes for transplants, implants, grafts and artificial devices, which imply an underlying disease. The WICC Task Group had alternatively recommended: (i) Retaining the traditional Class for general multi-system primary prevention activity in Chapter A, as in ICPC-1 and ICPC-2, (ii) retention of the separate “no disease” class for distinguishing administrative encounters which were not strictly prevention, and (iii) adding a new Class for preventive activity in each body system Chapter to define preventive activity associated with a specific body system but not associated with a specific disease (such as avoidance of risk of cardiovascular or sexually transmitted disease). With such a change, preventive activity for a specific disease would have been coded by using a new class for “management plan” in the Process component to qualify planned interventions for a specific disease. Such a solution would have added useful granularity, retained useful information about the purpose of preventive activity and complied with the traditional structure and rules of ICPC-1 and ICPC-2.

Chapter II is the category for “Functioning and Functioning Related” Classes. The Chapter code is now a Roman numeral, in contrast with the convention for most other ICPC-3 Chapters. In implementing this new Chapter, all ICPC-1 and ICPC-2 Classes which defined disability in each body system, in each Chapter, both as an RfE and an episode title, have been deleted. Such constitutes a formal split between function and disease, with one Chapter for the former and the remaining Chapters for the latter. Thus, in ICPC-3, function is now categorised as a separate concept distinct from any symptom, disease or health problem. Consequently, each RfE and diagnosis coded with ICPC-3 must now be matched in some alternative way with all appropriate functioning codes, unless the RfE or diagnosis is exceptionally considered to have absolutely no effect on function, and vice versa. This constitutes a complete reversal of the standard ICPC convention without empirical data or formal academic support. Classically, with ICPC one codes the RfE as presented, and the diagnosis as made, and consequently one only codes disability as an RfE when so expressed, and/or codes a diagnosis of disability when such was considered to be the appropriate episode title. In ICPC-3 this process will be replaced by laborious (and subjective) double-coding of the RfE, or the EoC, linked to each and all Chapter II Classes which should be elicited through directed questioning to quantify functioning. The Cartesian dualism of the mind–body dichotomy is effectively internalised as a core element of ICPC-3. This dualistic conceptual fracture is also the major reason that ICPC-3 is inconsistent with, and does not fully map to, most other standard international classifications and coding systems in medicine, including ICPC itself and ICD. ICPC-3’s Chapter II constitutes a departure from the biological, psychological and social conceptualisation of illness and disease, since it splits function from disease. The WICC Task Group had recommended retaining the codes for disability and problems with functioning in each ICPC Chapter, thus retaining the link between a disability or functioning problem with a body system or systems, and then using a linked scale or tool to measure functional status.

The Intervention or Process codes, which comprised the second to sixth Components of ICPC-1 and ICPC-2 and were included in each Chapter, are now split off as a new ICPC-3 Chapter I.^9^ Chapter I alphanumeric codes follow a different convention from other ICPC-3 Chapters, with three-digit numbers, where the first digit defines membership of a sub-section (termed “sub-component”) reflecting historical ICPC-1 and -2 Components (2 to 6), renumbered from 1 with a new sub-component (3) for “Programmes related to reported conditions.” Adding the first letter of an ICPC-3 Chapter to the three-digit code specifies localisation information for the Intervention or Process, or else “A” can be used when more than one Chapter (body system) is involved. Still, when localising to Chapter A1 (no disease), two letters are to be appended to the code (“AF,” “AG,” “AI,” “AP,” “AQ” or “AR” as appropriate for the respective sub-components of Chapter A1), resulting in a five-digit code for Interventions or Processes nominally not performed for an associated disease. This creates a minor inconsistency in ICPC-3 code length. Additionally, not all the possible combinations of A1 sub-components and Interventions or Processes make clinical sense (for example “AI106” would code for a urine test for patient introduction or treatment preference, “AF114” would code for an electrical tracing for family planning, etc.), and it is not desirable to have such inappropriate codes. The exponential increase in granularity consequent to the profusion of new Classes created using this system will almost certainly result in Rubrics with very low or zero prevalence, with a consequent reduction in precision and reliability.

### Prevention classes

The *aim* of a defined preventive activity together with the *intervention* performed are often both included in Chapter A1 Rubric labels for prevention Classes and in Chapter I Intervention and Process Class Rubric labels. Besides the unnecessary duplication, this means that for the first time ICPC prevention and Process Classes will be specific to a defined and limited context. Any additional detail provided by such a change in ICPC-3 was already available in ICPC-1 and ICPC-2 through the joint use of separate and specific codes for the RfE (including requests for interventions), intervention and episode title, both for “no disease” (primary) prevention and for interventions related to prevention of an existing disease or problem. Thus, coding the Process or Intervention linked with a code for the actual disease or problem gave context to both elements in turn both in ICPC-1 and ICPC-2. Therefore, the change in ICPC-3 does not, in fact, add new granularity *per se*. Such simply adds context descriptions to both prevention and Process Class labels, to no obvious advantage since the purpose of the Process and prevention labels is to describe the content of the class, and context can be otherwise provided, as above. This increased specificity simply limits Classes to specific contexts in ICPC-3. The WICC Task Group made an alternative proposal to add additional granularity to prevention Classes, through the expansion of the general prevention Class with sub-classes, or the addition of “no specific disease” prevention Classes in each ICPC-3 Chapter to code special primary prevention programmes for a body system (such as immunization to prevent a specific genitourinary disease without that disease being present). An alternative proposal to add context information to specific Intervention and Process Classes was made by the Process Group.

### Process classes

Chapter I “Interventions and Processes” Class labels now often include the process description as well as its purpose or scope, or name the target disease (see above). As explained previously, this apparent increase in specificity actually only limits usability. This change, together with the transition from universal Components to a single Chapter I, has simply created structural tensions and historical incongruences without advantage (see footnote 9). The alternative solution proposed by the WICC Process Group, and approved by the Task Group, was to create a new Process Class for formal management programmes. This would have allowed the coding of preventive programmes implemented for specific diseases, or for general preventive purposes, defined by additionally coding the appropriate disease or “no disease” Class, respectively. In such case, linking the preventive programme code with the specific examination, investigation and medication Process codes (including the specific Chapter alpha) would have substantially expanded granularity, linked each to a specific disease or body system, and defined that each was performed as part of a formal preventive programme. This innovative system would have been fully consistent with ICPC-1 and ICPC-2, substantially more specific, applicable in all contexts, and would not have required structural changes to the classification.

### Functioning and functioning-related classes

Chapter II “Functioning and Functioning Related” is intended for coding function or disability. Classes in subcomponent 2F0 “participation and activities” can be exclusively used to code either a RfE of EoC, whilst they, together with other sub-components, can additionally be used to measure and code functions, personality functions and environmental factors. Thus, functioning is categorised as a separate body system, or distinct problem area, in ICPC-3, and its Classes replace the traditional −28 ICPC Class^10^ which was present in every body system Chapter. Consequently, function classes in ICPC-3 lack localisation information, which can only be coded by linking such codes to other ICPC-3 codes separately. Additionally, such a Chapter split between functioning and disease creates an internal fracture (see above). Chapter II Classes can also be used as an instrument for measuring and coding function and disability, which is new for ICPC-3. However, the units of measurement of severity have not been tested against a gold standard, and the interpretation of such data is still undefined. The WICC Task Group had decided on the retention of a − 28 Class in each ICPC Chapter, thus maintaining the excellent capability of ICPC to code disability either as an RfE or an episode title, localised to a body system. The Task Group recommended linking such Classes to a validated tool to allow the assessment of functioning, disability and health to the required level of detail. With all its new Classes, ICPC-3’s Chapter II loses localisation information with the lack of specific body Chapter alpha codes, and the added value of the new quantification system is unclear without validation.

Chapter II did not emerge from empirical primary care data, and consequently does not follow the granularity limits applied to all other Classes during the revision process by the WICC Chapter groups. Chapter II Classes derive from an entirely different healthcare paradigm, comprising a sub-set of 52 codes selected directly from the International Classification of Functioning, Disability and Health (ICF) (11) by an expert group, including members of the ICPC-3 Consortium. The selection emerged from existing chronic disease ICF subsets, and was originally designed as a self-administered questionnaire for assessing functioning in Dutch patients with chronic conditions. This questionnaire was *internally* validated in focus groups of approximately 30 Dutch patients with chronic disease, and subsequently in a questionnaire study on 565 patients, without testing against existing gold standards (12, 13). During ICPC-3 development, Consortium members agreed to add concepts from another independently developed French tool, “Arrêts de Travail en médecine générale à partir de la Classification Internationale de Fonctionnement’ (ATCIF),” similarly derived from ICF for the purpose of assessing functioning in French primary care patients on sick leave (14), and additionally from the WHODAS 2.0 scale (15). Chapter II thus merges three sub-sets of ICF, independently derived for quite different purposes than to order the domain of family practice. These three instruments have never been combined before, and the first two have not been externally validated either singly or in combination. It is also unclear how a tool or tools designed as a set of concepts, scored singly and added together to measure functioning, can be broken down so that its items are then used individually. Such a scoring system is thus not a classification of the components of functioning in primary care. The analysis and interpretation of such data will be challenging, since this implementation is in conflict with the international standard application of ICF.

Indeed, the official ICF manual specifies that the coding system is designed for profiling patient functioning, or measuring health in general, using pre-selected sets of codes qualified numerically, with at least one qualifier needed per code (to define categories or to scale severity). Such ICF sub-sets should be validated by field trials across populations. The interpretation of such scores (typically expressed as a 0–100 disability scale), require the derivation of meaningful cut-off thresholds from empirical data. Consequently, the use of existing validated ICF-based tools, such as WHO’s WHODAS 2.0 (15), is highly recommended. Moreover, it is explicitly stated that ICF is complementary to clinical terminologies and classifications, and should be linked to them for joint use, since ICF scores usually require a clinical diagnosis to facilitate interpretation. In fact, ICF is most often used with ICD, but is certainly not included within the core ICD structure. Additionally, the manual lists known limitations of ICF, including the lack of a clear ontological structure, failure to resolve conceptual ambiguity, and misalignment with standard clinical terminologies (16). Essentially, the literature defines ICF as a scoring system to be used with, and not within, clinical terminologies or classifications.

Most ICPC-3 codes are nominal (present/absent) whilst the Chapter II codes have ordinal values.^11^ Thresholds for these ordinal categories remain undefined, with negative implications for coding and analysis. This combination of nominal and ordinal data impacts any mathematical operation, including calculating such basic epidemiological parameters as incidence and prevalence.

Chapter II Classes not only fail to resolve ambiguity between themselves, as in ICF (11, 16), but additionally overlap conceptually with other existing ICPC-3 Classes in other Chapters (e.g., FS05 “Decreased visual acuity” and 2F01 “Watching,” or HS02 “Hearing complaint” and 2F02 “Listening”). Should this be intentional, in splitting off and distinguishing function from disease, the duplication of some such symptoms but not others cannot but reflect an inconsistent implementation of such across Chapter II Classes.

### Miscellaneous issues

The inclusion of concepts from a broader domain, specifically functioning and health measures derived from ICF, lacks a formal academic base. This decision to include measures of functioning and health also contrasts with the decision to not include Classes for clinical signs and medication, preferably as an extension. The ICPC-3 revision was performed without the required intermediate step of a formal foundation layer of concepts, which would have facilitated the identification and resolution of ambiguity across categories and concepts. This would also have allowed the implementation of an upper limit of granularity, and ensured homogeneity of such across all Classes.

Usability of the new system will pose challenges due both to Chapter labels and Rubric codes which are alphanumerically inconsistent, as well as Rubric codes of varying length which are frequently ordered and numbered counter-intuitively. Such risks alienating users familiar with ICPC-1 and ICPC-2 codes, which could have been avoided with less extensive re-ordering. The inclusion of regional extensions, with Rubrics for Classes of even higher granularity, poses additional challenges for internationally consistent data collection and analysis.

The revision process has also introduced grammatical and spelling errors, as well as imprecisions in the descriptions, inclusion and exclusion texts. A standardised labelling system, proposed by WICC and formalised by the WICC Task Group, as well as a Rubric Tag system for meaningful groupings (e.g., infections, injuries, neoplasms, etc.) have not been implemented.

Users of ICPC-3 may have to obtain a separate licence to use ICF, since this is now included (at least in part). ICPC-3 includes descriptions of Classes, many of which deviate from international standard definitions. This could be problematic should such be construed as definitions which may have a perceived or actual negative impact on patient care.

The incompatibility with all existing classifications and coding systems, as well as the non-standard implementation of ICF, will challenge users who have to use any such systems as part of their daily work. The content of the doctor-patient consultation in primary care will be significantly affected by the use of a coding tool consisting of over 90 items to measure functioning, with the risk of incomplete data entry during time-limited consultations.

## Proposed solutions

In the two formal reports forwarded from the WICC Task Group to the ICPC-3 Consortium (Appendices 1, 2), alternative solutions to the issues which arose during the development of ICPC-3 were presented, in the first instance (Appendix 1) as proposals to adhere to standard classification principles, and in the second instance (Appendix 1) (Appendix 2) during our critical review of the actual published version of ICPC-3. Some of these solutions are also detailed, or referred to, in the article text above. In summary, we would recommend that ICPC-3 be urgently revised to implement the following major changes to address the most critical problems above, including the process of revision itself:

- Governance issues should be resolved to harmonise workflow between WICC and the Wonca-ICPC-3 Foundation. Authorship issues should be resolved, appropriately assigning intellectual property and formally recognising it. The editorial control of ICPC-3 should be assigned to the full WICC Committee, and delegated appropriately. Membership of ICPC-3 task groups should be open to all WICC members, based on sound academic background and technical expertise.
- A robust structure for ICPC-3 should be re-defined
- Including functioning as an axis or extension, not as a Chapter but as a validated assessment tool linked to the core of ICPC-3 using the Class for “disability” (RfE or problem) as a placeholder or link to that extension in each Chapter
- Creating axes or extensions for coding drugs, laboratory and imaging tests, and clinical findings with the appropriate placeholders or links
- Creating appendices for personal factors and severity indices
- Clarifying rules for the use of indices/scales, extensions and function Classes
- Adopting, or modifying, existing reliable and validated coding systems or scales for the above purposes, or creating new ones as appropriate, as an ongoing process
- Documenting all changes in a continuous log file with appropriate version control.
- Revising ICPC-3 Classes should now involve
- Revising all Rubrics for consistency with defined principles and rules, especially mutual exclusivity
- Re-numbering in a standard way with minimal departures from historical Rubrics
- Re-assigning family planning and prevention to their appropriate Chapters. Re-assigning prevention activity as a Class in each Chapter
- Re-organising Chapter G with due consideration of sex-linked and non-sex-linked rubrics
- Improving the consistency of Process Rubric code letters and numbering. Revising Classes following the Process Group recommendations, including to avoid including context in a Rubric label and adding a Class for managed programmes
- ICPC-3 Classes should be mapped to equivalent or related Classes or codes in major alternative Classifications or coding systems
- Reviewing Rubric labels for consistent formatting. Errors in definitions, inclusion and exclusion criteria and English spelling should be identified and resolved
- Submitting the finished product to external peer review, and formal empirical testing.

The ICPC-3 Consortium has now been dissolved, and ownership and responsibility for all three ICPC versions has been devolved to a new Wonca-ICPC-3 Foundation. It is hoped that such changes may be discussed and implemented in future within this new framework.

## Conclusion

ICPC has traditionally been characterised by domain completeness, mutually exclusive Classes, a single terminology for the patient’s reason for encounter and the family doctor’s diagnosis, and Classes defined according to international standards and emergent from empirical primary care data. ICPC follows strict frequency limits and taxonomic rules, including the prioritisation of localisation over aetiology and the use of symptom diagnoses to appropriately define and measure diagnostic uncertainty. Such has meant that ICPC has naturally become the international standard primary care classification, fully compatible with, and yet complementary to, other classifications and coding systems, whilst offering a unique perspective on patient’s symptoms and requests and their relationship with disease and health problems.

The publication of ICPC-3 in December 2020 finalises a process whereby the ICPC-3 Consortium deviated from formal decisions made by WICC, the author of ICPC, and its members. Many such changes defy categorisation and conceptualisation standards, and thus render ICPC-3 significantly incompatible with existing international coding systems. ICPC-3 now represents an untested departure from international standard presentations, without a formal academic base. Amongst other serious errors, the direct inclusion of measures of functioning in a classification of reasons for encounter and health problems fails to address the dichotomy of these domains, the boundaries of and relationships between which are not satisfactorily resolved by the system. Rather, such ambiguity is a burden transferred to ICPC-3 users, and to the patients whose data would thereby be captured. Analysis of these data will require the development and implementation of alternative, as yet undefined, models of the relationships between disease and health, which, ironically, cannot be informed by ICPC-3 data due to the inherent challenges of analysis. By including different domains without resolving ambiguity, and by splitting function from other body systems, ICPC-3 becomes an internally fractured instrument.

## Data availability statement

The original contributions presented in the study are included in the article/supplementary material, further inquiries can be directed to the corresponding author.

## Author contributions

JKS led the writing of the manuscript and the review of ICPC-3. NB, EC, TF, SV, and MU participated in the writing of the manuscript and the review of ICPC-3. All authors contributed to the article and approved the submitted version.

## Footnotes

^1^An Episode of Care, as distinct from an episode of illness or disease, is a health problem or disease from its first presentation to the health care provider, to the last presentation for the same problem.

^2^A code or Rubric in ICPC defines one concept, a symptom or sign, intervention, problem or disease.

^3^The reason(s) for encounter (RfE(s)) is defined as an agreed statement of the reason(s) why a person enters the health care system, representing the demand for care by that person.

^4^ https://www.globalfamilydoctor.com/groups/WorkingParties/wicc.aspx

^5^Chapters are defined by a letter (alpha) and are based on body systems or health problem areas. Rubrics in each chapter are divided amongst seven Components defined by two-digit number ranges: Component 1 (symptom- and sign-label Rubrics numbered 1–29); Component 7 (disease-label Rubrics numbered 70–99); Components 2 to 6 (process-label Rubrics numbered 30–69). Exceptionally, some disease-label Rubrics were historically put in Component 1 in Chapter S (Skin) rather than Component 7, due to space limitations. Over time it was agreed to correct this, and also to move specific disease-label Rubrics from the cardiovascular to the neurological Chapters in the next revision to follow changing international consensus, besides other similar changes.

^6^These roles and relationships were formally defined in 2016, and were formally re-confirmed at the WICC meetings in 2017 (France) and 2018 (Ukraine).

^7^Although a minority of WICC members were also members of the Consortium Core Group and Taskforce, they did not report formally back to WICC on many important discussions or decisions until they were finalised.

^8^Such was expressed either through e-mail communications on the official WICC e-mail list server, or more formally at the WICC meetings in 2019 (Crete, Greece) and September 2020 (Germany).

^9^Chapter I classes are grouped as a Chapter, and as such are presented differently from Components in ICPC-1 and ICPC-2. This is in itself a significant change, as now Interventions and Processes, together with function, count as Chapters, whereas in ICPC-1 and ICPC-2 Chapters were reserved for body systems.

^10^In ICPC-1 and ICPC-2, the concept of generalised “Limited function/disability” or “Limited function/disability [of a specific body system]” was coded with a single −28 Rubric in Component 1, with the appropriate chapter alpha as its first element, to be used to code an expressed RfE or the title of an EoC as appropriate.

^11^In ICPC-1 and ICPC-2 the coding of a class indicates presence and null (no code) indicates absence. In ICPC-3 many rubrics (especially in Chapters II and V) have numerical qualifiers, with a zero value for absence and a range or values coding a qualifier such as severity. Chapter II codes used as an RfE or EoC will require further qualification of severity during coding.
