# Supplementary material for: The fractured lens: a controversial revision of the International Classification of Primary Care
Source: Front Med (Lausanne). 2024 Jan 11;10:1230987. doi: 10.3389/fmed.2023.1230987 (PMC10808642; doi:10.3389/fmed.2023.1230987)
Supplement: Supplementary file 1 [file Data_Sheet_1.docx]

# International Classification of Primary Care Principles and rules

Wonca International Classification Committee ICPC-3 Task Group
Jean K Soler (Lead), Elena Cardillo, Nicola Buono, Olawunmi Olagundoye, Dimitris Kounalakis, Gojo Zorz

Version 01.02 (March 2020)

# Introduction

The development of the two current, and third emergent, revisions of the International Classification of Primary Care (ICPC) was, is and remains a complicated and multi-faceted process. It requires rigorous academic work to produce a valid and reliable classification capable of representing the content of international primary care.

The development of ICPC-3 continues, being led by a Consortium of stakeholders, implemented by experts at the University of Nijmegen and guided by the Wonca International Classification Committee (WICC) through a specific Task Group created therein.

The Task Group first reviewed and harmonised earlier work by Chapter Groups, tasked with reviewing each ICPC Chapter, and has now been tasked with reviewing all the changes in ICPC-3 implemented by the Consortium.

The WICC Executive Committee has agreed that the Task Group should now formalise a list of rules and principles to guide the ongoing development of ICPC-3. These rules are to be presented to and discussed with the Consortium.

The aim of the process is to make cooperation between the WICC Task Group and the Consortium more formal and transparent, and to allow a more formal, semantically and structurally consistent development of ICPC-3, compliant with an explicitly defined set of principles and rules.

# Foundation layer (Structure and Content)

ICPC-3 has been developed using ICPC-2’s concepts and structural framework as its original basis and foundation. The ICPC-3 foundation layer, which is currently in development, has evolved almost exclusively from ICPC-2 concepts and structure, albeit with significant changes which were formally agreed within WICC.

New additions have emerged from other classifications or terminologies mapped to ICPC, specifically ICD-10, ICD-11 and, to a much lesser extent, ICPC-2-Plus. Deletions have also followed the rules applicable for ICPC-2. Other changes have been proposed reflecting terminologies and coding systems which are not based on mutually exclusive classes, and are not constrained within the domain of primary care, such as SNOMED-CT and ICF. Such latter changes may present a challenge unless formal principles and rules are defined for changes to ICPC, specifically for ICPC-3.

A large proportion of the work of the Chapter Groups, tasked with reviewing every ICPC chapter, and the process group( tasked with reviewing the ICPC process codes), has been based on the discussion of ICPC-2 rubrics, and supported with data collected using ICPC-2. Decisions to expand and collapse rubric content have also been based largely on ICPC-2 data. As such, the principles and rules of ICPC-2 have implicitly been incorporated into ICPC-3 through this evolution. Additionally, the very name of ICPC-3 reflects that it is an evolution of ICPC-2.

Such an evolution means that the development of ICPC-3 was evidently not a *de novo* process. The implicit rules of ICPC-2 were not always formally and explicitly defined in the past, but were largely followed by the Chapter groups (WICC members reviewing each ICPC Chapter) and the Task Group in the process of suggesting and reviewing the changes above. Within the Task Group it was agreed to follow the rules of ICPC-2 by default, save for justified and agreed exceptions, which are duly reasoned. Such a course of action has the support of many WICC members.

This document shall formalise principles and rules as an essential part of the work in developing ICPC-3. Without such rules and principles, the development of ICPC-3 risks becoming less consistent, valid and reliable.

**Principles and rules:
1. ICPC-2 was the source of the emergent foundation layer, concepts and structure of ICPC-3.**

**2. ICPC-3 concepts emerge largely from the review and re-organisation of ICPC-2 concepts.**

**3. During this development process, the basic principles and rules did not change, with some exceptions.**

**4. The major agreed changes in moving to ICPC-3, decided by WICC, included:**

**a. the modified structure of the codes to create more space.**

**b. the merging of Chapters X and Y.**

# Structural integrity

ICPC, in revisions 2 and 3, is a classification, and as such follows all the rules for a classification, in the domain of primary *care*.

*Classification:*

“the act or process of putting people or things into groups based on ways that they are alike”
<https://www.merriam-webster.com/dictionary/classification>

“the systematic organization of knowledge by classes, structured hierarchically according to subdivisions defined by attributes”

*Class / Category*:

“A set of objects grouped on the basis of similarity of attributes selected as significant according to the purpose/s of the classification activity”

Each class can in turn be divided into smaller sets (subclasses) according to further defining criteria.

**Principles and rules:**

1. **ICPC aims for domain completeness, and covers the whole domain of *primary care.***
2. **ICPC is designed to record all the elements of the encounter in *primary care*, that is: the *reason/s for the encounter, the process of care (intermediate and resulting interventions),* and the *diagnosis.***
   1. ***ICPC does not include classes for clinical findings, even though some symptom or disease label classes are defined by a clinical-finding label.***
3. **ICPC rigorously includes only mutually exclusive classes, with residual classes to keep the main classes clean.**
4. **ICPC is empirical, and classes emerge from actual incidence and prevalence data from primary care.**
5. **ICPC is designed for international use, and should be appropriate for primary care in all countries, at least those represented in Wonca.**
6. **ICPC is divided into chapters, which represent body systems or areas of primary health care.**
7. **ICPC is bi-axial, and the second axis is represented by components:**
   1. **Components 1 (symptom labels) and 7 (disease and problem labels) are both used for labelling the diagnosis.**
   2. **Components 2 to 6 (process labels) are all used for labelling intermediate and resulting interventions.**
   3. **Components 1 to 7 are all used to label reason/s for encounter (including requests for interventions).**
   4. **The number of components can usefully be collapsed to three: symptom label, process label and disease label, defined by rubric second alpha characters “S”, “P” and “D” respectively. The historical sub-division of process codes can instead be incorporated as an archetype tag (see below).**
8. **ICPC follows the hierarchy of Chapter, Component (some common across all Chapters, some unique to each Chapter), and Rubric (i.e. class). The core classification should retain this structure:**
   1. **To maintain international representativeness and relevance, regional extensions may be linked to ICPC in this latest revision, specifically where additional granularity is desirable due to the higher regional prevalence of symptoms or diseases which are not present in the core international classification:**
      1. **The historical and preferred method to achieve this aim is through the mapping of an ICPC class to appropriate classes in the International Classification of Diseases (ICD).**
      2. **However the creation of one or more sub-classes, to be used only in a defined region, should be allowed as long as all the ICPC rules and principles are followed. Such sub-classes should exclusively be sub-classes of a single parent class, and such sub-classes should (together) contain the entire content of the parent class (if necessary with the inclusion of a residual class ((rag-bag)), which is almost always required). Each sub-class inherits the characteristics of the parent class and can also have additional features/attributes. Such sub-classes should be an extension of the core of ICPC, but should never be included in the core, to avoid the creation of an additional hierarchal level which is not consistently applied across the entire classification. One example could be the regional extension of a rag-bag for infections, so to include viral infections with increased regional prevalence. However, this can also easily be addressed by using ICD as an extension, as with the ICPC-2-ICD-10 mapping.**
      3. **The use of the inclusion criteria to include a list of concepts which are hyponyms or sub-concepts of the class is desirable, but such inclusion criteria text should not be linked to codes or sub-codes (see “8” and “8.a” above).**
   2. **The need for increased granularity for other reasons should also be addressed by mappings to other classifications or coding systems.**
9. **Localisation takes precedence over aetiology.**
10. **ICPC does not cater for the mind-body dichotomy, and does not assign an aetiology to syndromes or symptoms.**

**Proposed major changes:**

**The number of components can usefully be collapsed to three: symptom label, process label and disease label, defined by rubric second alpha characters “S”, “P” and “D” respectively. The historical sub-division of process codes can be incorporated as an archetype tag (see below).**

**To maintain international representativeness and relevance, regional extensions may be linked to ICPC in this latest revision, specifically where additional granularity is desirable due to the higher regional prevalence of symptoms or diseases which are not present in the core international classification.**

# Content

The following general principles apply to the International Classification of Primary Care as a whole, and define its content:

**Principles and rules:**

1. **Empiricism: based on clinical data from primary care.**
2. **Domain completeness: there is a place for everything.**
3. **Mutually exclusive concepts: everything in its place, one place.**
4. **The classes should resolve ambiguity, should not include parent/child categories (super-concepts and sub-concepts) at the same hierarchical level, and should include residual classes to keep the major classes clean (by capturing data which is not better classified by the major classes).**
5. **The granularity should be appropriate and justified with empirical data. A prevalence of at least 0.5 per 1000 patient years (0.05%) is required to justify a separate class, for a classification which can reliably reduce the clinical content of observations from a single primary care practice of around 2000 patients over one year.**
   1. **Such prevalence should be considered both from the perspective of the reason for encounter *and* the problem label.**
   2. **When it may be considered desirable to sub-divide a class and split out a new class (or indeed to add sub-classes or a hierarchical level for sub-classes (also see point “8” above”)), notwithstanding marginal prevalence data to support this split (for example, due to concepts which are politically important, have major public health importance, or changes to address semantic requirements (e.g. consistency or subsumption)), the major considerations should be that:**
      1. **both the granularity and**
      2. **the structure (i.e. the hierarchical level/s) should be consistent across the classification**
      3. **that any consideration of adding other hierarchical levels or sub-divisions should also follow the rule of a consistent minimum frequency**
      4. **and that decisions regarding additions and/or deletions of classes with marginal prevalence should be consistent across the Chapters and classes.**
   3. **In the case of persistent doubt, the decision taken for ICPC-2 should take precedence to maintain historical consistency.**
6. **ICPC is calibrated on data collected using the *episode of care* model. This model allows for improved precision of incidence and prevalence measures for conditions which may either present multiple times as separate episodes, or as part of one or more multi-encounter episodes.**
7. **Individual decisions to move (across chapters), merge or split rubrics should be:**
   1. ***individually* justified by frequency data and**
   2. **consistent, both internally within ICPC and externally with other international standard classifications.**
8. **Consistency with international standards for defining and categorising diseases, such as the *International Classification of Disease (ICD),* the *Diagnostic and Statistical Manual (DSM)* and *Snomed-CT.* This holds especially for recent changes occurring in the evolution from ICD-10 to ICD-11.**
9. **Rubric codes are mnemonic to facilitate manual coding.**
10. **Changes should be exclusively justified by the following questions:**
    1. **Does the proposal align with the structure of ICPC?**
    2. **Does the proposal align with the principles of ICPC?**
    3. **Does the proposal fit within the classification rules (including semantic rules)?**
    4. **Is the proposal supported by frequency data?**

**In summary, changes to ICPC in the current revision should reflect revised information on incidence and prevalence (for the splitting or collapsing of classes), revised definitions of the content of a class, or revised conceptualisation of a class which changes its appropriate place within the hierarchy (specifically, cross-Chapter changes) . The more major the change (such as the creation of a high level hierarchical sub-division or collapse, for example at Chapter level) the more justification and consensus should be required.**

# Archetypes and rubric tags

The “colour codes” historically included in the paper version of ICPC-2 were useful for grouping classes according to specific aetiological attributes, such as ‘infections’ or ‘neoplasms’. However, incorporating grouping attributes in the rubric code itself would have the effect of adding another axis, or component, or structural element which would itself also have to follow the rules of non-ambiguity.

Many classes may have different aetiologies, such as pleurisy, which may be neoplastic, infectious, iatrogenic, traumatic, etc.. Assigning membership to only one group, exclusively, would be an inappropriate reduction. Such would mean that, for example, a disease can be an injury but cannot be an infection, or vice versa. It is not desirable to solve this fracture by creating different classes for different aetiologies of a class at the same hierarchical level (for example, neoplastic pleurisy and infective pleurisy, at the same hierarchical level as pleurisy with undefined aetiology). ICPC prioritises localisation over aetiology, but still takes into consideration the aetiology, reflected in the numbering sequence and the colour codes. ***This double characteristic of ICPC needs to be represented properly when creating a new revision.***

An alternative proposal is to allow for different grouping attributes to be assigned to each class, and this is facilitated with an archetype model. Such grouping attributes may then be used for easy and appropriate grouping of concepts. These grouping attributes may be identified by the first letter of the group name, such as “N” for “neoplasm” or “I” for “infection.” Archetypes, distinctly from colour codes, allow each class to possess different attributes, or belong to different groups, simultaneously. Thus, the multiple aetiologies of classes, such as pleurisy, can be easily represented for grouping purposes, or to collect additional information about aetiology in an individual patient. In the case of classes with multiple attributes, the user should be encouraged to code the attribute, for additional precision. Alternatively, hierarchical expansion using mapped classifications such as ICD can help resolve additional detail on aetiology.

Other uses for archetypes and rubric tags are given below.

**Proposals:**

1. **Grouping of risk factors for retrieval, mapping, to facilitate user coding or to add individual case detail:**
   1. **Grouping of all neoplasms (possible sub-grouping into malignant, benign and other/undefined/unspecified).**
   2. **Grouping of all infections (possible sub-grouping into viral, bacterial, fungal, parasitic).**
   3. **Grouping of all injuries.**
   4. **Grouping of all congenital diseases.**
   5. **Grouping of process codes reflecting the historical 2 to 6 subdivisions which may now be collapsed.**
   6. **Other groupings, similar to the original colour codes, but taking advantage of the flexibility of such tags to avoid errors which may have been committed in the past.**
2. **Identifying rubrics which should be used only in males, or females, or in defined age groups.**
3. **Labelling diagnoses which are usually only incident once (i.e. life-long), to facilitate error-trapping during coding.**
4. **Alternative views for the new AANN code structure, to allow shorthand codes similar to ICPC-2 codes (e.g. RS05 / R05S / R05 can all be linked to the concept “cough”, with “RS05” as the primary rubric code and the others as rubric tags).**
5. **Interoperability:**
   1. **Embedded links to thesauri.**
   2. **Mappings to other classifications.**
   3. **Other interoperability applications.**
6. **Indexing and information retrieval purposes (although this function may be more appropriate for the interface terminology which is planned to be built and linked to ICPC-3).**
7. **The archetypes and rubric tags are *not* a new axis or component, and so do not cause problems of ambiguity.**
8. **The number of components can usefully be collapsed to three: symptom label, process label and disease label, defined by rubric second alpha characters “S”, “P” and “D” respectively. The historical sub-division of process codes can be incorporated as an archetype tag.**

# Renumbering

Historically, ICPC-2 was easy to use since codes were easy to remember, with mnemonic Chapter alphas and mnemonic number sequences, with similar concepts (such as “pain” or “infections”) at similar places in the numbering sequence of each Chapter. With the new structure, a change in numbers will dis-empower users who code manually, or use three-key shortcuts in their electronic medical records.

**Principles and rules:**

1. **Number (two-digit) changes should be kept to an absolute minimum.**
2. **As far as possible, the new codes should only incorporate changes in the second letter (second alpha), and not the two-digit code. Changes in the alpha and numeric components of ICPC rubrics should be minimised in the revision, to retain the skill-set of current users.**
3. **The order of concepts should not be changed arbitrarily, for the same reason.**

**Proposal:
Space may be created in all components, with minimal changes in the numbering:**

1. **The process codes may be renumbered separately from other ICPC-3 codes, or else retain the same ICPC-2 numerical digits. However, the proposal is to recode the process codes with an “*α*P” prefix (“*α*” being the ICPC-3 Chapter, e.g. “*S*P01”), and starting with number -01. This will retain the Chapter link, as with ICPC-2, and also allow the “P01” style abbreviation (without the Chapter alpha, equivalent to the convention in ICPC-2, e.g. “-30”) for grouping data on processes across Chapters. It is recommended to use a dash (e.g. “-P01”) to avoid confusion with the ICPC-2 codes in Chapter P.**
   1. **By subtracting 30 units from each code number, for example (“-31” becomes “-01”), it will be easy to remember the new numbers (“A62” will become “AP32”, “D45” will become “DP15”, and so on).**
2. **The space created by freeing the numbers 30-69 may be used to extend the two-digit codes of the 1^st^ and 7^th^ component in each chapter:**
   1. **The Component 1 ragbags and “fear of” rubrics could be re-numbered to reserved numbers -41 to -49 in each chapter. Thus Component 1 shall have reserved numbers -01 to -49 (20 additional spaces, per chapter).**
   2. **The Component 7 codes may be renumbered -50 to -99, possibly with the use of the new (smaller numeric value) codes (-50 to -69) for less common disease-label rubrics, such as neoplasms of congenital malformations or even infections (19 additional spaces, per chapter). An alternative case could be made to shift all disease-label codes up by a fixed value (for example, re-start at -50) so that the numeric change is easier to remember (e.g. R74 – URTI becomes RD54 – and all other codes shift up by a fixed value of 20, like with the process codes above).**
   3. **Such would allow the creation of space to accommodate new rubrics (only 30 or so are required, all over ICPC-3), whilst allowing the old numbers to be retained for most rubrics. The numeric changes will involve less-commonly used rubrics, and most rubrics that are used frequently will retain their old numbers.**

# Prevention

The prevention and “no-disease” codes are highly prevalent. A proposal to add granularity in this area has wide support.

This is already possible in ICPC-2 with post-coordination. If all elements of the encounter are coded, the content of the episode of care of preventive activity is more clearly defined. Thus, an episode of “A98 - Prevention” associated with a Reason for Encounter “A44 – immunization” and with an intermediate intervention “A44” clearly describes an episode of care of vaccination, which can then be further categorised with the actual vaccine given, coded with the WHO ATC system. Such rich information cannot be incorporated into a single code, especially considering the current ICPC structure and rules.

Pre-coordinated classes will create issues with ambiguity, and this will be very difficult to manage otherwise. As such, the issue with prevention and the “no disease” category is best address by training users to code appropriately, and also by adding detailed information about post-coordination in the notes associated with the equivalent “A97” and “A98” rubrics in ICPC-3.

However, the splitting of the prevention class is justified by frequency. This is best achieved by putting a class for prevention in each ICPC-3 Chapter.

**Proposal:**

1. **Creating a new chapter must follow all the ICPC rules, including having/accommodating 7 components, being appropriate for both RfE and Problem labelling, empirically supported by data from primary care, consonant with the rules of mutual exclusivity and domain completeness, and justified by frequency. The proposal for a new chapter on Personal Factors, including prevention and risk factors, is not consonant with these rules. It also deviates from the principle of using Chapter A as the default residual Chapter. It should not be implemented in ICPC-3.**
2. **To improve coding of preventive activity, suggestions for users to appropriately code requests for and actual interventions (possibly included in the notes or comments of A97 and A98) should be added to ICPC-3 prevention classes.**
3. **Having a code for prevention in each Chapter would to improve the granularity of data on prevention, especially when the RfE is not coded. *This is the preferred solution.***
4. **The idea of sub-codes (children) of the prevention rubric is also to be considered, but must respect all the structural principles and rules.**
5. **Risk factors can be coded with the archetype tags proposed above, which avoids the need for grouping in a separate Chapter. A discussion on which disease-label concepts are diseases and which are simply risk factors (e.g. diabetes type II, hypertension, hyperlipidaemia, osteoporosis, etc.) shall then be necessary.**

# Clinical findings

ICPC-2 was designed to code reason/s for encounter, interventions and diagnoses (episode titles) with component 1 (symptom labels), 2 to 6 (process labels), and 7 (disease or problem labels). ICPC-2 does not cater for coding clinical findings.

However, some classes in ICPC-2 had labels which represent a clinical sign (e.g. fever, high blood pressure), when such is used to describe a symptom (e.g. fever) or a problem label (e.g. high blood pressure). These are not clinical signs *per se*.

In SNOMED-CT, ***all*** symptoms are coded as findings (observations), since symptoms are not accommodated. This is the exact opposite approach with respect to ICPC-2. For example, in SNOMED-CT the symptom “fever” is coded as “fever (finding).” To be technically correct, in SNOMED-CT you should measure the temperature and find it to be high before using the code “fever (finding)”. In order to code the symptom “fever” presented by the patient, you should double-code the “fever (finding)” with the SNOMED-CT code for “context: reason for encounter.” Such a system is incompatible with ICPC, at the fundamental level, and cannot be correctly mapped. If SNOMED-CT does not code symptoms, no mapping with any other classification will resolve this major limitation.

If the proposal to move rubrics with the label of a clinical finding to Component 1 is followed through, the rubric for “Death”, which is clearly a clinical finding, would also have to move to Component 1. The moving of component 7 classes to component 1 on the basis of their label (specifically, referring to a clinical event) will lead to inconsistencies, and should not be pursued.

It is incorrect to state that ICPC-2 or ICPC-3 incorporates a coding system for clinical findings. Such a coding system could be, or maybe should be, developed by WICC, and this has been proposed by the Process working group. Such a future development is to be recommended, but the publication of ICPC-3 should not be delayed by such a process.

**Proposal:**

1. **Do not incorrectly refer to component 1 (symptom label) or component 7 (disease label) classes as clinical findings, since this is incorrect. ICPC codes symptoms, diseases and interventions, and not clinical findings.**
2. **Keep with the original ICPC-2 convention for rubrics labelled with a clinical finding term:**
   1. **Symptoms labelled with a clinical finding term should be in Component 1 (e.g. lump, fever, cough).**
   2. **Problems labelled with a clinical finding term should be in Component 7 (e.g. high blood pressure, abnormal enzyme levels, death).**
3. **Develop a classification for clinical findings in primary care, in future.**

# Standardisation of labels and accompanying text

The labels of most of the Rubric components have changed from ICPC-2 to ICPC-3. Such changes now need to be justified, and rules and definitions of each element should be provided.

Within the Task Group, consensus has formed that it is necessary to have strict rules for formatting the text labels of Rubrics.

**Proposal:**

1. **The rules for the ICPC-3 labels should be documented, discussed and implemented:**
   1. **Component 1 specifically:**
      1. **where the lay and the clinical term are synonyms, they both should be included in the rubric label with a slash between them: e.g.: haematemesis/vomiting blood;**
      2. **where the lay term is a hypernym or hyponym of the clinical term, the lay term(s) should not be in the rubric label, but should be included in the 'Inclusions' section. However, it was historically accepted that this may not always be the best solution, and that if it *must* be included in the rubric label, the two concepts should be separated by a semicolon, rather than a slash, in the rubric label;**
   2. **Both component 1 and 7:**
      1. **the rubric label should not include sub-parts of the rubric. Sub-parts (hyponyms) should be in the Inclusions, *not* in the rubric label: e.g. “*Condition* acute/chronic” from ICPC-2, should now be labelled “*Condition*” in ICPC-3 (with "acute" and "chronic" in the Inclusions). However, this does not apply should two different classes (e.g. different Rubrics for "acute" and "chronic") be appropriate.**
2. **The concepts and the actual titles of the “inclusions” and “exclusions”, “considerations”, “notes” and “ICD-10” mappings have not been carried over faithfully from ICPC-2 to ICPC-3. All changes must be justified, with appropriate references and/or documentation. It is proposed that the original ICPC-2 terms, definitions and rules have stood the test of time, and should not be changed without justification. The practice of referring to the standard classifications and definitions of diseases (ICD and DSM) should be retained.**

# Fear and functioning

-26 – “fear of cancer…” and “-27 fear of other…” are consistent across chapters, and there have not been many calls for major change. However, it is a cross-chapter issue, and should be reviewed by Task Group A, in the next phase of its work

-28 – limited functioning/disability was frequently used in some chapters, much less so in others:

- as a RfE: A28 – 0.6/1000py; B28 – 0; D28 – 0.1; F28 – 0.3; H28 – 0.1; K28 – 0.1; L28 – 4.9; N28 – 0.6; P28 – 0.1; R28 – 0.2; S28 – 0; T28 – 0; U28 – 0.2; W28 – 0.1; X28 – 0; Y28 – 0; Z28 – 0.1 (Dutch Transition Project data, rates per 1000 patient years);
- combined RfE + Problems: A28 – 0.5/1000py; B28 – 0.2; D28 – 0.4; F28 – 1.5; H28 – 0.5; K28 – 0.8; L28 – 0.4; N28 – 0.5; P28 – 0.2; R28 – 0.1; S28 – 0; T28 – 0; U28 – 1.2; W28 – 0.1; X28 – 0.4; Y28 – 0.2; Z28 – 1.9 (ditto).

Thus, removing all -28 codes from all Chapters is not justified by the frequency data.

**Proposal:**

1. **Creating a new chapter must follow all the ICPC rules, including having/accommodating 7 components, being appropriate for RfE and Problem labelling, based on empiricism, consonant with the rule of mutual exclusivity and domain completeness, and justified by frequency. The proposal for a new Chapter on Functioning is not consonant with these rules.**
2. **A classification of functioning and disability already exists and is maintained by WHO (ICF). There is no need for WICC to create a new coding system for this purpose. The existing classification includes qualifiers for indicating severity of the disability or impairment, for the localization and the nature, etc.. Including parts of this classification in ICPC-3 is not appropriate, since concepts either do not fit the existing Chapters and their symptom, intervention, or problem components, or break the rule of mutual exclusivity due to overlap with existing ICPC rubrics. One must consider that ICF is complimentary to ICD-10, and that WHO encourages their use together (ICD-10 classifies diseases and comorbidities, ICF classifies states of health and functioning). In all utilisation cases, the health condition (disease) is coded with ICD-10, and then the functioning is coded with ICF. Thus, even in the case of ICPC-3, the use of a classification of functioning should be complimentary, and not included in the core. If ICF concepts are included in the core, their mapping to ICD-10 will be problematic.**
3. **It is proposed that all existing rubrics for “fear of” and “limited functioning” remain in Component 1 in each Chapter, as in ICPC-2. Having codes for limited functioning in each Chapter helps one understand the actual disability, not just as a functional problem, but understanding which body system is affected.**
4. **A classification of functioning and disability could be adopted (the preferred choice is ICF), modified, or created by WICC and mapped appropriately to the appropriate ICPC-3 rubrics. Such a classification could additionally address such issues as severity, temporality and other related factors. It should be considered that such attributes would also apply to related areas not directly linked to functioning, such as other symptom-label concepts in Component 1.**

# Joining chapters X and Y, and making Chapter W unisex

Chapters X and Y will be joined as Chapter G, to remove the sex-linked codes in ICPC-3. The justification for this decision was that information regarding patient sex is available from the medical record, and thus does not need to be captured by the classification. However, the reason for separating rubrics in the sex-linked Chapters in ICPC-2 was more to do with the actual nature of sex-linked diseases and symptoms, which are innately linked to the patient’s sex.

Thus, despite the proposed change, many rubrics in the proposed new Chapter G will still be innately sex-linked (such as “prostate cancer,” “penis pain,” “vulval pruritus” and “ovarian cyst”). As such, the join does not resolve the sex linkage issue. Rubrics will not be unisex. Similar issues will arise in Chapter W, and in fact may be much more problematic since most concepts are inherently sex-linked.

This creates a major fracture, with many sex-linked (male or female) symptoms but more unisex disease concepts. The analysis of diagnostic associations will require post-hoc sex identification of male and female patients labelled with the disease, since the classification alone will not be able to resolve this. The analysis of the diagnostic association between “breast lump –female”, and “breast cancer – female”, will be impossible in ICPC-3 unless the data has a separate tag for male and female patients, or is linked to other data from electronic health records which allow determination of the subject’s sex. The diagnostic association between a symptom and a genital disease may be quite different for the two sexes, and it is essential to retain the capability to make such an analysis. In situations where such information is not reliably available, ICPC-3 cannot be used to study such diagnostic associations, or to reliably measure the prevalence of sex-linked diseases.

Once you lump you cannot split. However, we propose a better way to reduce the sex-linkage of genital diseases which are not substantially different in the presentation in different sexes

**Proposal:**

1. **One position supported in the Task Group is to keep the original ICPC-2 Chapters W, X and Y. It would be possible to only join those rubrics in Chapter X and Y which are genuinely unisex.... such as “syphilis,” “gonorrhoea,” and “herpes.” These could be moved to Chapter A or Chapter U, even though they fit better in Chapter X/Y/G. However, this proposal has been previously rejected by the full WICC Committee.**
2. **An alternative solution, supported by the Task Group, is to merge Chapters X and Y to create a Chapter G with three alphas available for each rubric (“G” or “X” or “Y”) depending on the following rules:**
   1. **“X” for those rubrics which are exclusively female;**
   2. **“Y” for those rubrics which are exclusive to males;**
   3. **“G” for unisex rubrics.**
3. **With this proposal, it is also possible for the user to be offered the choice of using different letters in different contexts:**
   1. **“G” being most appropriate in datasets with available patient sex identifier data;**
   2. **“X” and “Y” being most appropriate when sex identifier data is not available, and so must be captured using the Classification.**
   3. **For this to work, such “joined” codes will need to have the exact same two-digit code, so that the rubric meaning would not be changed by assigning “X”, “Y” or “G”. This is the same as using process codes without a chapter identifier (e.g. -31) for grouping purposes, with the “G” being the grouping of the two sexes just as the “-“ allowed grouping of process codes across chapters .**
4. **Chapter W should remain sex-linked, since the concepts within are innately linked to the female sex.**

# Personal and risk factors

The issue of personal factors emerged from small group discussions in WICC. Factors such as age, sex, gender, height, ethnicity, social status, hobbies, habits, religion, occupation, etc. are not captured by ICPC-2. Such is correct and consistent, since such factors are not part of the domain of primary care, and especially not part of the reason for encounter, intervention and diagnosis model of the encounter. As such, they are not part of the core of ICPC-2, and should not be included in the core structure of ICPC-3 without a change in the fundamental rules of ICPC. However, such a formal rule has never been proposed to, or adopted by, WICC. In fact, the discussions have always leaned towards a separate coding system for personal factors

Personal factors should be, and often actually are, recorded in other parts of the medical record, and separately retrieved for analysis as appropriate. When such personal factors actually are an RfE or a problem, then they should be double-coded with both ICPC-3 codes and personal factor codes (e.g. age and age-related dementia; financial problems (as an RfE) and poor socio-economic status). This is equivalent to the current situation in ICPC-2, when personal factors which were presented as a RfE or became a diagnosed problem, were coded with ICPC-2, but other personal factors were not.

It is a procedural fracture to exclude sex (a personal factor) from ICPC-3 by joining “X” and “Y” into Chapter “G” but then introduce personal factors as part of the Social Chapter “Z”.

The move of certain rubrics from the General Chapter “A” to another location inn ICPC-3 is also inconsistent with the rule that Chapter “A” is the residual chapter for those rubrics which are not linked to a body system.

The grouping of risk factors in ICPC-3 will be easier should the archetype proposal be implemented. As such, the need to move risk factors to one Chapter will be eliminated. It is desirable to have risk factors in appropriate Chapters, as in ICPC-2, but now also to have the facility to label a condition (either a symptom or a disease) as a risk factor, using the archetype model.

**Proposal:**

1. **Personal Information and Personal Factors are not to be included in the core structure of ICPC-3. They should not be coded with ICPC-3 codes unless they are presented as a RfE or diagnosed as a Problem. This is already the case with Chapter Z in ICPC-2, and this approach should be retained.**
2. **A classification of personal factors needs to be adopted, modified or created by WICC and mapped to appropriate ICPC-3 rubrics. This coding system should capture data on factors influencing health including social, environmental and personal factors, occupation, hobbies and habits, health approach, and possibly attitudes, religion, spirituality and belief.**
3. **All risk factors should be grouped using archetype tags. It is not useful to remove risk factors from each of the main ICPC Chapters, simply in order to group them.**

# Review of code changes implemented in ICPC-3

Up till the September 2019 WICC meeting in Crete, the following changes were recommended by the Task Group

1. Many changes to inclusions, exclusions, considerations, and notes.
2. Major rubric changes:
   1. 4 rubrics to change chapter, 4 to consider changing chapter;
   2. 8 code deletions, with 2 more to consider;
   3. 24 new codes, possibly another 7 if frequency data supports this;
   4. 5 codes to be merged into another, with 1 more to consider.

**Proposal:**

**All changes actually made to ICPC-3 by the Consortium working group should be reviewed by the Task Group. Since there is, unfortunately, no version control for the Consortium work process, this will require a separate fixed dataset to be created. Such a version would allow review of all rubrics by the Task Group, without changes being made by other users of the ICPC-3 browser.**
