# Supplementary material for: The fractured lens: a controversial revision of the International Classification of Primary Care
Source: Front Med (Lausanne). 2024 Jan 11;10:1230987. doi: 10.3389/fmed.2023.1230987 (PMC10808642; doi:10.3389/fmed.2023.1230987)
Supplement: Supplementary file 2 [file Data_Sheet_2.docx]

# International Classification of Primary Care Version 3

## Review of ICPC-3 stable version (version April 2020)

Jean Karl Soler, Elena Cardillo, Nicola Buono, Dimitris Kounalakis and Gojo Zorz for the
Wonca International Classification Committee ICPC-3 Task Group

Version 01.01 (July 2020)

# Introduction

The development of the two current, and third emergent, revisions of the International Classification of Primary Care (ICPC) was, is and remains a necessary, but complicated and multi-faceted process.

It requires rigorous academic work to develop and/or revise a classification which is a valid and reliable reduction of the content of international primary care. The validation of the classification, in particular, requires openness to peer review to identify and address errors and inconsistencies.

The development of ICPC-3 continues, being led by a Consortium of stakeholders, implemented by experts at the University of Nijmegen and guided by the Wonca International Classification Committee (WICC) through a specific Task Group created therein.

Much important progress has been made with the current revision: for example with new ICPC-3 classes; a new defined rubric code format; re-organisation of specific Chapters; resolution of various cross-Chapter issues; changes in the class descriptors, criteria and the mappings to other classifications and terminologies; the accommodation of region-specific classes; and a new approach to the assessment of functioning. A lot of work has gone into defining all ICPC-3 classes and mapping pre-existing, and the, classes to various international classifications and nomenclatures. However, not all such changes have emerged from decisions, or even discussions, within WICC, and some present inconsistencies with, or departures from, the principles and rules which have defined ICPC for decades.

The WICC ICPC-3 Task Group (Task Group Alpha; TGA; also referred to as Task *Force* Alpha) first reviewed and harmonised earlier work by the Chapter Groups and the Process Group within WICC, which were originally tasked by WICC with the review of each ICPC-2 section. As part of this process, a document on the principles and rules which define ICPC, and how they should or could be applied to ICPC-3, was produced. The WICC TGA is now tasked by the Wonca Europe Executive Board with reviewing all the changes in ICPC-3 implemented by the Consortium.

The aim of the process, and this document, is to make cooperation between the WICC Task Group and the Consortium more formal and transparent, and to allow a more formal, semantically and structurally consistent development of ICPC-3. This review shall assess whether the ICPC-3 current revision is compliant with an explicitly defined set of principles and rules, and specifically those which have been defined in a document (International Classification of Primary Care Principles and rules Version 01.02 (March 2020)) already sent to the Consortium. Solutions shall be proposed for any identified issues.

It is the impression of the TGA that no other formal report on the defining principles and rules of ICPC has been formally compiled specifically to inform the development of ICPC-3. As such, this work is considered to be fundamental to an internally consistent, valid and reliable revision of ICPC-3.

# Wonca International Classification Committee

The Wonca International Classification Committee is the author of ICPC, whilst Wonca retains publisher rights and copyright of the work, and licenses it. Wonca and WICC have devolved the task of developing ICPC-3 to a time-limited Consortium. Work on the ICPC-2 codes to inform the development of ICPC-3 started within the ICPC-2 Update Group which reviewed ICPC-2 data to identify improvements necessary to maintain ICPC, and also to inform the future development of ICPC regarding necessary changes to be implemented in future revisions. Much work on ICPC-3 has been done within specific Chapter Groups (including the Process Group) within WICC, which have reviewed all ICPC-2 codes, referencing available datasets, and proposed changes to be implemented in ICPC-3. Many such changes have been approved by full WICC discussions and votes. Within WICC, a specific Task Group (Task Group Alpha, TGA; originally named as such when formed at the WICC Lyon meeting in 2017; sometimes called Task *Force* Alpha) was tasked with harmonising the feedback from the Chapter Groups and reviewing all cross-chapter changes and issues to inform the revision of ICPC-3.

The relationship between these entities was defined by numerous unanimous decisions during official face-to-face WICC meetings, and was formally listed in the minutes of the meeting in Lviv, Ukraine, in August 2018, such minutes being approved formally at the meeting in Heraklion, Crete in 2019:
“**I. Governance and editorial control of ICPC**

1. The ICPC works are ultimately “owned” by Wonca

2. Wonca have divested the functions of editorial control of ICPC products to WICC, including structure and content

3. WICC have embarked on the creation of a new product, ICPC3

4. WICC retains editorial control over ICPC3

5. WICC (and Wonca and Wonca Europe) have together created and assigned a new entity, “the Consortium”, to help with the timely completion of ICPC3

6. The consortium in doing their time-limited work, will contribute elements of structure and content to ICPC3. In other words the consortium will share authorship.

7. Editorial control over ICPC3 remains with the following caveats:

a) The Consortium is expected to deliver the ICPC3 product in a timely manner. It is important and reasonable that they receive deliverables of content and structure according to a list of agreed deadlines and milestones.

b) In the event of failure of WICC to deliver a) above, the Consortium should assume editorial control for that deliverable, subject to reasonable review by WICC.

c) In the unfortunate situation where there are undue delays or failures of agreement between WICC and the Consortium, the Wonca executive will need to adjudicate, as owners of ICPC products.

**II. Processes within WICC to support the production of ICPC**

1. Chapters of ICPC3 have been allocated to chapter groups to look at each chapter to suggest alterations and additions/deletions to chapter content/structure

2. Revised chapters will be passed to Task Force A (TFA), whose principal function is to look for and fix cross chapter interactions and anomalies.

3. Task force A will then deliver completed the deliverable work packages to the Consortium in accordance with the milestones and deadlines.

4. In the event of any problems in this workflow, issues will be taken up with WICC executive.

5. It is to be expected that on inspection and processing by the consortium, new issues of structure and content will arise. WICC must devise a rapid mechanism to accept these changes.

**Ultimately Wonca executive may choose to intervene to resolve undecided delayed issues. This would be an unfortunate and possibly existential issue.”
(Official WICC minutes Ukraine 2018)**Such is also evident from the role definitions, goals and objectives formally published on the official Wonca WICC page (<https://www.globalfamilydoctor.com/groups/WorkingParties/wicc.aspx> accessed May 2020):

“The mission of the WONCA International Classification Committee is to develop and maintain classifications that accommodate the complete domain of family/general practice, and to ensure that these classifications are interoperable to the highest degree possible with standard international health care terminologies and classifications, in order to contribute to equitable quality health care worldwide.”

“From the mission statement, we derive the following primary goals:

- To achieve widespread international use of the International Classification of Primary Care (ICPC)
- To maintain and revise ICPC to accommodate expanded health care knowledge
- To develop productive working relationships with other international standards development organizations
- To support the work of WICC and WONCA through licensing of ICPC
- To create and disseminate additional classification tools as needed to capture and codify the complete domain of family/general practice

These goals lead to the following core objectives for the work of WICC:

- Stabilize ICPC-2 in electronic and print form (version control)
- Reconcile and maintain maps between ICPC and ICD, ICPC and SNOMED
- Complete licensing agreements for ICPC-2 in new countries, particularly developing areas
- Identify a stable and sustainable Web-based host for ICPC-2
- Develop a training program for ICPC
- Create and maintain a professional Web presence for WICC
- Promote additional translations of ICPC and other primary care classification tools
- Create a “primary care classification package” of ICPC-plus-other terminologies built in to simple open-source health IT intended for use in developing areas
- Build the conceptual foundation for ICPC-3 (the “primary care data model”)
- Complete collaborative agreements with WHO and IHTSDO
- Identify additional classification needs and develop new tools to fill those needs
- Build capacity for data analysis and field trials within WICC or WONCA
- Strengthen working relationships with WONCA Informatics Working Party
- Create an international “Primary Care Classification Consortium” to organize and fund our activities”

Within the Task Group Alpha (TGA), the original guiding principles for revising rubrics in ICPC-3 were defined in January 2018 as follows:

“• Does the proposal align with the structure of ICPC?

• Does the proposal align with the principles of ICPC?

• Does the proposal fit within the classification rules (including semantic rules)?

• Is the proposal supported by data on frequency of term usage?”

As such, the decisions within WICC, the Chapter Groups, and the TGA have always supported the retaining of the structure and principles of ICPC, as implicitly incorporated in the first (ICPC-1, 1987) and second (ICPC-2, 1998) revisions. Such principles and rules have been extensively described in the original ICPC books of the same dates, numerous international standard publications, and have now been summarised and extended with the view of developing ICPC-3 in a document produced by the TGA (International Classification of Primary Care Principles and rules Version 01.02) in March 2020.

# Authorship and Governance

Unfortunately, this issue has been problematic of late.

WICC has repeatedly confirmed, unanimously, that authorship of ICPC-3 resides with WICC itself. The Consortium was originally created to implement the technical work in revising ICPC-3, and to follow decisions made by WICC or delegated to the Task Group Alpha within WICC (see above).

Wonca and the Consortium should immediately issue a clear and binding statement on whether the authorship of ICPC-3 shall be that of WICC. Whilst the Consortium Partners should be duly recognised for their work, WICC cannot be denied authorship due to the heavy overlap between the current revision of ICPC-3 and previous versions. In default, the voluntary work by members of WICC, who have subsidised Wonca with their time, expertise and, often self-funded travel and accommodation, will be de-valued and orphaned. Any publication of ICPC-3 without the authorship of WICC and those members who have contributed over the years should not even be considered, let alone proposed or implemented. The Consortium’s role should be that of a publisher, and individuals within the Consortium should be listed as co-authors if they have significantly contributed to the content.

Hand in hand with authorship lies control of content. The current situation where the Consortium has a separate task group, which is working independently from WICC, creates problems with effectively exercising authorship rights and obligations. Such becomes more acute, and important, when work performed separately by the Consortium task group involves methodologies which are not aligned with official WICC positions, or even not made known to WICC, documents are produced but not shared, changes are made to an online ICPC-3 version but they are not logged, etc.

It is inevitable that two scientific groups working separately will have different considerations and outcomes with respect to the new resource, but such should lead to further discussion of these different outcomes. Recently, such discussion is being curtailed or avoided. This is especially true should WICC or TGA reports on such changes be ignored or shelved.

The current situation is that there have been no formal requests for review of Consortium proposals to either WICC, the WICC Chapter Group leads, the WICC Process Group or the WICC TGA. Consortium documents have been presented as final versions, the ICPC-3 Browser does not allow direct commenting, and there is no formal log of changes made on the road to ICPC-3 (let alone requests for feedback on such). It is not possible for users to access various ICPC-3 versions, or to download intermediate versions from the website. Therefore, collaborative review by actors not directly involved in the Consortium is very difficult. Many attempts to provide such feedback have been met with resistance from the Consortium task group.

There is no formally published academic basis or data source for much of ICPC-3. The data sources for many new classes are not documented. Even though the approach and preliminary foundation model have been presented at WICC meetings over the last two years, there is no formally published ICPC-3 foundation model or layer (except for a short description and a figure provided in one informative page on the Consortium website), with a defined conceptual framework to allow the hierarchy and categorisations to emerge empirically therefrom. There is no ICPC-3 rule-book, or even a list of formal definitions. There is no register of datasets or literature reviewed, or of the analyses performed. There is no documentation of the data which support each new class, or its estimated prevalence. There is no formal, publicly available summary of the discussions which support, or do not support major changes, let alone direct WICC or TGA participation in such discussions.

In summary, besides the original documentation which was an outline of the work process, no scientific or technical reports or documentation have been formally produced and shared by the Consortium, not even a documentation of the work itself. The recent detailed documentation submitted by the TGA to the Consortium has not elicited an item-by-item reply, and the request for a frozen version of ICPC-3 as an offline dataset, for TGA to review and comment on, has not been accepted. Even though the Consortium has requested a detailed class by class review of ICPC-3 via the browser, such a task appears intricate and time consuming, especially considering the short time available for the task by TGA volunteer members. Now, this task could be performed, once the TGA work in other areas is nearing completion. However, the position taken by the Consortium is that such a review will not be considered.

This is not an ideal state of affairs, and is not conducive to an open development process for ICPC-3.

**Recommendation:**

**The authorship and editorial control of ICPC-3 should remain with WICC, and the relationship between Wonca, WICC and the Consortium should follow the guidelines issued by WICC and unanimously agreed upon. Such authorship shall not deny co-authorship or acknowledgment of those who have contributed financially or scientifically to the development of ICPC-3.**

**A new working relationship should be defined to avoid the duplication of efforts and the lack of effective leadership which have emerged from the recent work processes. Issues identified by WICC and the TGA should be discussed by the Consortium and detailed and formal responses to each proposal, or problem identified, should ensue, with a view to resolving such issues. A register of changes to ICPC-3 will allow better understanding of the changes implemented, allow documentation of supporting data, and facilitate academic review.**

# Timeline of WICC work supporting the revision of ICPC

In order to resolve any issues with authorship of ICPC, the following is an abridged timeline of contributions that WICC and its members have made to the development of ICPC, and specifically its current third revision:

- Annual meetings, mostly subsidised by Wonca member organisations of self-funded by Wonca members, since the 1970s
- Publication of ICPC-1 in 1987, including a reference book publication
- Publication of ICPC-2 in 1998, including a reference book publication
- Publication of ICPC-2R (Revised) in 2005, including a reference book publication
- ICPC-2 Update Group meetings in April 2011 in Amsterdam, the Netherlands, June 2011 and March 2014 in Malta, and October 2012 in Ravello, Italy
- First review of ICPC-2, with the development of the methodology for reviewing rubrics to inform ICPC-3 development, in Malta in June 2011
- Chapter Group formation, and review of all ICPC-2 rubrics by Chapter at the WICC meeting in Lisbon, Portugal in September 2014
- Chapter Group work between 2014 and 2019, on all ICPC Chapters and rubrics
- First formal proposal for the Consortium and of the ICPC-3 TGA in Turku, Finland in September 2016
- Formal institution of the TGA at the WICC meeting in Lyon, France in 2017
- Finalisation and submission of TGA review of all 17 Chapter Group submissions by Chapter, 2018 to 2019
- Formal detailed TGA feedback to Consortium on original Content Model documentation in May 2019
- Feedback of TGA and WICC members to Consortium on ICPC-3 changes implemented to date at Crete meeting in September 2019
- Process Group review of Process codes in ICPC-3 at the WICC meeting in Crete in September 2019, conclusion via Skype meeting in April 2020 and final report reviewed by TGA and being submitted to the Consortium
- Formal detailed TGA feedback to Consortium on ICPC-3 principles and rules in March 2020
- This document, a review of the current ICPC-3 revision and its conformity with established ICPC principles and rules

# Foundation layer (Structure and Content)

ICPC-3 has been developed using ICPC-2’s concepts and structural framework as its original basis and foundation. The ICPC-3 foundation layer, not formally published, has evolved almost exclusively from ICPC-2 concepts and structure. Many ICPC-3 concepts are *de facto* actually also ICPC-2 concepts, and the overlap between the two is very high. As such, it is not clear how the principles and rules for ICPC-3 have been re-defined or modified, or what the new rules and principles are.

A large proportion of the work of the Chapter Groups, tasked with reviewing every ICPC chapter, and the Process Group (tasked with reviewing the ICPC process codes), has been based on the discussion of ICPC-2 rubrics, and supported with data collected using ICPC-2. Decisions to expand and collapse rubric content have also been based largely on ICPC-2 data. As such, the principles and rules of ICPC-2 have implicitly been incorporated into ICPC-3 through this evolution. Additionally, the very name of ICPC-3 reflects that it is an evolution of ICPC-2.

Such an evolution means that the development of ICPC-3 was evidently not a *de novo* process, even though the approach has changed. All the rules and principles of ICPC are defined in the ICPC books published through Wonca. These books, and related publications, were referenced by the Chapter Groups (WICC members reviewing each ICPC Chapter) and by the TGA in the process of suggesting and reviewing the changes above. Within the TGA it was agreed to follow the rules of ICPC-2 by default, save for justified and agreed exceptions, which are duly reasoned. Such a course of action has the support of many WICC members.

The major changes agreed by WICC in moving from ICPC-2 to ICPC-3 included the renumbering to create new space, the merging of sex-linked codes into one chapter, and the retiring and inclusion of rubrics based on a defined frequency threshold. Issues such as functioning, risk factors and personal factors were extensively discussed, but there was no formal agreement to depart from the established models in ICPC-2, mainly because alternatives remained problematic and controversial. As such, there was never a formal WICC decision to ignore, retire or fundamentally change the principles and rules governing ICPC, especially not to accommodate new inclusions.

**Guiding principles:
1. ICPC-2 was the source of the emergent foundation layer, concepts and structure of ICPC-3.**

**2. ICPC-3 concepts emerged largely from the review and re-organisation of ICPC-2 concepts.**

**3. During this development process, the basic principles and rules did not change, with some exceptions.**

**4. The major agreed changes in moving to ICPC-3, decided by WICC, included:**

**a. the modified structure of the codes to create more space.**

**b. the merging of Chapters X and Y.**

**Review of ICPC-3:**

1. **The foundation layer for ICPC-3 was not formally drawn up and distributed. This would have allowed many issues with structure, content and categorisation to be resolved at a higher level, with a more global view of the impact of such decisions.**
2. **Chapter A1 merges routine examination, prevention, family planning and other reasons for encounter. It is not clear how this Chapter is to be formally distinguished, at a conceptual level, from Chapter A, the general chapter, and possibly Chapter W which includes pregnancy and ante-natal care, both of which remain in ICPC-3. This change creates a problem with the mutual exclusivity of Chapters.**
3. **Chapter II contains Functioning and Functioning Related concepts. Functioning is an attribute of various body systems, and, furthermore, is not necessarily a problem. As such, assessment of functioning is better included in a separate axis (or extension, or mapping), as was the case with ICPC-1 and ICPC-2 and the Wonca Co-Op charts, and is currently the case with ICD (International Classification of Diseases) and ICF International Classification of Functioning, disability and health). Removing problems with functioning from the other body systems (and areas of health care, i.e. the other Chapters) creates an inconsistency with labelling such function problems, expressed either as a Reason for Encounter or Problem/Disease Label, separately in each single ICPC-3 Chapter. This approach is also inconsistent with other classifications included in the WHO-FIC.**
4. **Emergency codes are contained in a separate Chapter IV. This creates an overlap with other Chapters which would be better containers for an emerging urgent concept.**
5. **Regional extensions, scales, temporal measures and causality are included in Chapter V. Regional extensions should not have their own codes, unless the hierarchy of super and sub-concepts (with appropriate rag-bags) are appropriately handled within the new ICPC-3 structure. The original formal position by the Consortium was to include these extensions in the inclusion criteria of ICPC-3 rubrics. The inclusion of scales, temporal measures and measures of causality as a Chapter/s in ICPC-3 will create major issues with mutually exclusive concepts. As such, any such classes/measures should be included in a separate Axis/es or Extension/s.**
6. **Personal Factors, which could have been included in a new Axis or Appendix, have first been included and then removed from ICPC-3. This is a missed opportunity to address an important issue.**

**Recommendation:**

**The proposal is to include new Axes (or Extensions) for measures of Functioning, coding of Drugs, Laboratory and Radiological tests, and Clinical Findings; an Appendix for Personal Factors; and an Extension for Severity and Chronicity (acute/sub-acute/chronic/other) in a hierarchy which does not depart from the principles and rules of ICPC. Regional extensions should also not be part of the core of ICPC-3, and only published with regional versions of ICPC-3.**

# Structural integrity

ICPC, in revisions 2 and 3, is a classification, and as such follows all the rules for a classification, in the domain of primary *care*.

*Classification:*
“the act or process of putting people or things into groups based on ways that they are alike”
(<https://www.merriam-webster.com/dictionary/classification>, accessed May 2020)

“the systematic organization of knowledge by classes, structured hierarchically according to subdivisions defined by attributes” (<https://www.isko.org/cyclo/classification>, accessed June 2020)

*Class / Category*:
“A set of objects grouped on the basis of similarity of attributes selected as significant according to the purpose/s of the classification activity” (<https://www.isko.org/cyclo/classification>, accessed June 2020 )

Each class can in turn be divided into smaller sets (subclasses) according to further defining criteria.

Such division implies that each class/sub-class and group is distinct (thus, the division) and that each entity (person or object) will fit in one group according to a defined attribute. As such, mutual exclusivity of classes and domain completeness are ***defining characteristics*** of a classification.
 **Principles and rules:**

1. **ICPC aims for domain completeness, and covers the whole domain of *primary care.***
2. **ICPC is designed to record all the elements of the encounter in *primary care*, that is: the *reason/s for the encounter, the process of care (intermediate and resulting interventions),* and the *diagnosis.***
   1. ***ICPC does not include classes for clinical findings, even though some symptom or disease label classes are defined by a clinical-finding label.***
3. **ICPC rigorously includes only mutually exclusive classes, with residual classes to keep the main classes clean.**
4. **ICPC is empirical, and classes emerge from actual incidence and prevalence data from primary care.**
5. **ICPC is designed for international use, and should be appropriate for primary care in all countries, at least those represented in Wonca.**
6. **ICPC is divided into chapters, which represent body systems or areas of primary health care.**
7. **ICPC is bi-axial, and the second axis is represented by components:**
   1. **Components 1 (symptom labels) and 7 (disease and problem labels) are both used for labelling the diagnosis.**
   2. **Components 2 to 6 (process labels) are all used for labelling intermediate and resulting interventions.**
   3. **Components 1 to 7 are all used to label reason/s for encounter (including requests for interventions).**
   4. **In revising ICPC, the number of components can usefully be collapsed to three in ICPC-3: symptom label, process label and disease label, defined by rubric second alpha characters “S”, “P” and “D” respectively. The historical sub-division of process codes can instead be incorporated as an archetype tag (see below).**
8. **ICPC follows the hierarchy of Chapter, Component (some common across all Chapters, some unique to each Chapter), and Rubric (i.e. class). The core classification should retain this structure:**
   1. **To maintain international representativeness and relevance, regional extensions may be linked to ICPC in this latest revision, specifically where additional granularity is desirable due to the higher regional prevalence of symptoms or diseases which are not present in the core international classification:**
      1. **The historical and preferred method to achieve this aim is through the mapping of an ICPC class to appropriate classes in the International Classification of Diseases (ICD).**
      2. **However the creation of one or more sub-classes, to be used only in a defined region, should be allowed as long as all the ICPC rules and principles are followed. Such sub-classes should exclusively be sub-classes of a single parent class, and such sub-classes should (together) contain the entire content of the parent class (if necessary with the inclusion of a residual class ((rag-bag)), which is almost always required). Each sub-class inherits the characteristics of the parent class and can also have additional features/attributes. Such sub-classes should be an extension of the core of ICPC, but should never be included in the core, to avoid the creation of an additional hierarchal level which is not consistently applied across the entire classification. One example could be the regional extension of a rag-bag for infections, so to include viral infections with increased regional prevalence. However, this can also easily be addressed by using ICD as an extension, as with the ICPC-2-ICD-10 mapping.**
      3. **The use of the inclusion criteria to include a list of concepts which are hyponyms or sub-concepts of the class is desirable, but such inclusion criteria text should not be linked to codes or sub-codes (see “8” and “8.a” above).**
   2. **The need for increased granularity for other reasons should also be addressed by mappings to other classifications or coding systems.**
9. **Localisation takes precedence over aetiology.**
10. **ICPC does not cater for the mind-body dichotomy, and does not assign an aetiology to syndromes or symptoms.**

**Proposed major changes:**

**The number of components can usefully be collapsed to three: symptom label, process label and disease label, defined by rubric second alpha characters “S”, “I”(Alpha, not Roman Numeral; alternative “P” as second Alpha in an ICPC-3 code) and “D” respectively. The historical sub-division of process codes can be incorporated as an archetype tag (see below).**

**To maintain international representativeness and relevance, regional extensions may be linked to ICPC in this latest revision, specifically where additional granularity is desirable due to the higher regional prevalence of symptoms or diseases which are not present in the core international classification. All such inclusions should rigorously follow rules for creating sub-classes. Such extensions should not be part of the core or ICPC-3.**

**Review of ICPC-3:**

1. **Diagnoses and Reasons for Encounter (RfEs) can each be coded with a Component 1 (symptom label) or Component 7 (disease or problem label), and additionally component 2-6 codes may be used to code a request for an intervention as a RfE. As such, inclusion criteria are appropriate for guiding the choice of rubric to label a diagnosis or problem, both for Components 1and 7, and also for the process codes. Many Component 1 rubrics in many Chapters have either descriptions or inclusions, but not all have both.**
2. **Clinical findings are not coded in ICPC. Some Component 1 and Component 7 rubrics have the *title* of a clinical finding, but are used to code either a RfE or a Problem. It is confusing to describe Component 1 as including clinical findings. A classification of clinical findings is very desirable, and should be developed as a new Axis within or Extension of ICPC-3.**
3. **Overlapping concepts fracture a classification, which is built with mutually exclusive concepts which allow precise categorisations and reliable measures of incidence and prevalence. Examples of overlapping concepts in the current version of the ICPC-3 revision include:**
   1. **Sections AG, AI and AP which assign process labels to rubrics intended to label RfEs and health problems, thus creating pre-coordinated concepts and creating overlaps with process codes**
   2. **Chapter I sub-section 3, which assigns problem labels to process codes, thus creating pre-coordinated concepts and creating overlaps with the problem label**
   3. **Normal and abnormal functional status included in Chapter II sub-sections 2F and 2R being distinguished by a scale (0 represents normal). This overlaps with the ICPC-2 class A97 “no disease” and partially overlaps with the ICPC-2 class A98 “prevention,” both of which have been removed from ICPC-3 but have been replaced with new classes which also include the same concept of normality/no disease (e.g. AG03: Examination for certification purposes {i.e. with or without a disease} ) even though not exclusively! Additionally, the ICPC-3 rubrics in sub-sections 2F and 2R create an internal overlap between the presence of a problem and the absence of a problem within the same class. As such, ambiguity of disease/no disease is introduced in ICPC-3**
   4. **Various rubrics in Chapter II sub-sections 2F and 2R which overlap with social, neurological, hearing, locomotor, psychological and vision problems, pain, drug adverse effects, and other concepts appropriately coded in other ICPC-3 Chapters**
   5. **Chapter III comprising regional extension codes which overlap with ICPC-3 rubrics in other Chapters, and in fact share code number roots with those rubrics.**
   6. **Chapter IV emergency codes which will presumably include concepts which fit in other ICPC-3 Chapters**
   7. **Chapter V extension codes which include scales and attributes which apply to rubrics in other ICPC-3 Chapters.**
4. **ICPC has always been designed to code the Episode of Care over time, elegantly capturing the change of a problem label over time under the longitudinally continuous care of primary care providers. Such a concept is now an international standard presentation. The inclusion of a measure of the chronicity and acuteness of a condition in Chapter V must be implemented with clear guidelines which adhere to this international standard. Such measures of chronicity should preferably be implemented in a separate Axis or as an archetype/attribute, and not in a Chapter in ICPC-3.**
5. **ICPC has always preferred post-coordination, and avoided pre-coordinated terms. Examples of pre-coordinated terms in the current ICPC-3 revision include: processes with a defined purpose included in the label; prevention codes including a defined process in the label; and measures of functioning addressing a defined purpose, outcome or life situation. Such pre-coordinated concepts fracture the mutual exclusivity of ICPC-3 classes. Once the rules for a classification are not strictly adhered to, the argument for not including other post-coordinated terms becomes arbitrary. ICPC-3 seems to be neither a classification, nor a nomenclature, but a compromise between the two.**
6. **Chapter A1 combines prevention, family planning and other reasons for visit, but the conceptual distinction from Chapters A and W is not clearly defined. The removal of prevention from Chapter A and the removal of family planning from Chapter W will create significant differences with ICPC-1 and ICPC-2 Chapter incidence and prevalence distributions. The benefit of joining prevention, family planning and residual reasons for visit is not clear. The distinction between the concepts of the RfE and the “reason for visit” is not clear. The original RfE classes in ICPC-1 emerged from the American Reason for Visit classification, and as such they are quite similar, and in many cases identical.**
7. **Functioning is an integral part of each body system, and it is not clear how this is now a separate problem area, requiring its own Chapter. It is not clear how a lack of functioning of a body system can be coded, either as a RfE or problem label, in ICPC-3.**
8. **Regional extension codes are extensions of specific ICPC-3 rubrics. They could be offered as an alternative mapping from specified ICPC-3 parent classes, but should then follow all classification rules, including a residual class. As such these should not be included as a separate Chapter or Axis in ICPC-3, but should be mapped directly to their super- classes.**
9. **Emergency codes should not be in a separate Chapter or Axis, since they belong in their respective Chapters.**
10. **Scales could be a useful new Axis or Extension in ICPC-3. One example would be severity. However, strict rules should define how such would be used, especially how to apply such differentially to RfEs and episode or problem titles.**
11. **Chapter V also includes Causality. Such a concept should not be included in the core of ICPC-3, since ICPC prioritises localisation over aetiology. However, such aetiological tags may be usefully included as archetypes (see below).**
12. **Personal factors could be introduced as a new Appendix in ICPC-3.**

**Recommendation:**

**The proposal is to include new Axes or Extensions for measures of Functioning, coding of Drugs, Laboratory and Radiological tests, and Clinical Findings; an Appendix for Personal Factors; and an Extension for Severity and Chronicity (acute/sub-acute/chronic/other) in a hierarchy which does not depart from the principles and rules of ICPC (such as an archetype or rubric attribute, or possibly sub-classes for the different states {*the latter is the least preferred solution*}). The Chapter structure of ICPC should not be subject to major changes which are not empirically supported by robust data from primary care. Changes which disrupt the mutual exclusivity of classes should be addressed or reversed. Extension codes may be included as alternative mappings from specific super-classes already in ICPC-3, but only in regional versions.**

# Content

The following general principles apply to the International Classification of Primary Care as a whole, and define its content:

**Principles and rules:**

1. **Empiricism: based on clinical data from primary care.**
2. **Domain completeness: there is a place for everything.**
3. **Mutually exclusive concepts: everything in its place, one place.**
4. **The classes should resolve ambiguity, should not include parent/child categories (super-concepts and sub-concepts) at the same hierarchical level, and should include residual classes to keep the major classes clean (by capturing data which is not better classified by the major classes).**
5. **The granularity should be appropriate and justified with empirical data. A prevalence of at least 0.5 per 1000 patient years (0.05%) is required to justify a separate class, for a classification which can reliably reduce the clinical content of observations from a single primary care practice of around 2000 patients over one year.**
   1. **Such prevalence should be considered both from the perspective of the reason for encounter *and* the problem label.**
   2. **When it may be considered desirable to sub-divide a class and split out a new class (or indeed to add sub-classes or a hierarchical level for sub-classes {also see point “8” above”}), notwithstanding marginal prevalence data to support this split (for example, due to concepts which are politically important, have major public health importance, or changes to address semantic requirements {e.g. consistency or subsumption} ), the major considerations should be that:**
      1. **both the granularity and**
      2. **the structure (i.e. the hierarchical level/s) should be consistent across the classification**
      3. **that any consideration of adding other hierarchical levels or sub-divisions should also follow the rule of a consistent minimum frequency**
      4. **and that decisions regarding additions and/or deletions of classes with marginal prevalence should be consistent across the Chapters and classes.**
   3. **In the case of persistent doubt, the decision taken for ICPC-2 should take precedence to maintain historical consistency.**
6. **ICPC is calibrated on data collected using the *episode of care* model. This model allows for improved precision of incidence and prevalence measures for conditions which may either present multiple times as separate episodes, or as part of one or more multi-encounter episodes.**
7. **Individual decisions to move (across chapters), merge or split rubrics should be:**
   1. ***individually* justified by frequency data and**
   2. **consistent, both internally within ICPC and externally with other international standard classifications.**
8. **Consistency with international standards for defining and categorising diseases, such as the *International Classification of Disease (ICD),* the *Diagnostic and Statistical Manual (DSM)* and *Snomed-CT* is desirable*.* This holds especially for recent changes occurring in the evolution from ICD-10 to ICD-11.**
9. **Rubric codes are mnemonic to facilitate manual coding.**
10. **Changes should be exclusively justified by the following questions:**
    1. **Does the proposal align with the structure of ICPC?**
    2. **Does the proposal align with the principles of ICPC?**
    3. **Does the proposal fit within the classification rules (including semantic rules)?**
    4. **Is the proposal supported by frequency data?**

**In summary, changes to ICPC in the current revision should reflect revised information on incidence and prevalence (for the splitting or collapsing of classes), revised definitions of the content of a class, or revised conceptualisation of a class which changes its appropriate place within the hierarchy (specifically, cross-Chapter changes) . The more major the change (such as the creation of a high level hierarchical sub-division or collapse, for example at Chapter level) the more justification and consensus should be required.**

**Review of ICPC3:**

1. **The new Functioning Chapter includes classes which should satisfy the requirement for empiricism, and thus emerge from primary care incidence and prevalence distributions of RfEs and episode titles. The data sources used for the conceptual development of the classes should be quoted, and academic publications of the validity and reliability of the new classes, as well as the scale measurement (0-3; 0-4), should be provided for review. Such applies also if they have been outsourced from a different classification or coding system.**
2. **The conceptual model and categorisation process for the emergence of such classes should be explicitly defined. If a sub-set of the International Classification of Functioning, Disability and Health (ICF) is desired, the standard academic requirements must be satisfied, namely that such a selection of classes should emerge from an epidemiological study, and not simply an expert group selection.**
3. **ICF is not intended to label health problems and diseases, and as such cannot be included as a Chapter in ICPC-3, as such Chapters label RfEs, diseases and problems.**
4. **The Chapter II classes are coded with a scale (range 0-9), with zero defining the absence of a significant problem (therefore negative coding), 1-3 defining severity (with one additional sub-division creating a 4 point scale), and 9 coding “not applicable.”**
   1. **This system creates a major issue with a code for “present” and “not present” in the same class, distinctly from other ICPC-3 classes**
   2. **The optional three-point and four-point scales introduce ambiguity via such a choice, of a three-point or four-point scale. They also force a choice, even in the case of uncertainty, by imposing a scale rather than a code for presence versus absence. The latter four-point scale does not have a mid-point sub-class, whilst the former scale with three sub-classes also does not have a mid-point if you include the option to code “zero” (zero defining absence)**
   3. **It is unclear how the separate sub-divisions will be defined, across so many classes. What is a “complete” problem, and is such a definition valid and reliable, in primary care, across all the Chapter II classes?**
   4. **The intervals between 0 and 1, 1 and 2, 2 and 3, and 3 and 4, are unlikely to be homogenous or comparable, between themselves and, even more, across classes. How is this going to be catered for in any summative analysis?**
   5. **The residual sub-class (9) refers to “NOT applicable”, and thus does not adequately distinguish “not measured/unknown”, “uncertain”, “borderline” or a residual class (“other”)**
5. **The granularity limit (a prevalence of 0.5 per 1000 patient years) should apply to all the new classes, and data should be available to support the inclusion of all such classes (and the deletion of any class). This applies especially to the new classes in Chapter II, which have further subdivided classes which already did not have high prevalence in available ICPC-2 primary care datasets. This also applies especially for the scales in Chapter II, which further sub-divide such new classes.**
6. **A number of the new functioning classes in Chapter II directly overlap with symptom-label classes in the social, neurological, hearing, locomotor, psychological and vision Chapters, with classes for pain, drug adverse effects, and others.**
7. **There is some confusion in the label of Component 7 referring to it as including diagnostic codes (“Diagnosis/diseases”, *also inconsistent since the first is singular and the second is plural*). Component 7 includes classes with disease and problem *labels* which can be used to code a RfE (expressed as a problem or disease, e.g. “Doctor, I have come for my diabetes”) or a diagnosis. Component 1 (symptom label) classes may also be used to label a *symptom* diagnosis, and as such there is no “Diagnosis” component in ICPC.**
8. **The inclusion criteria for some component 1 rubrics are missing, and this may devalue their use as a code for a symptom diagnosis. Some rubrics have inclusions, and some do not; some rubrics have descriptions, and some do not.**
9. **There is no information on the estimated frequency of the included new classes. This is especially important as the TGA made specific recommendations that certain defined classes should only be included if the prevalence data indicated that they were observed as frequently than 0.5 times per thousand patient years of observation, or more.**
10. **The extensive renumbering ICPC-3 classes does not follow the mnemonic numbering system of ICPC-2, is inconsistent with the *two-alpha-two-digit* (2A2N) numbering system decided by WICC, and is also inconsistent with a four-character rubric code due to the two-character Chapter letters, three-digit rubric codes (process codes), scale extensions included in the code, and the various extension codes. It shall be practically impossible to recall ICPC-3 code numbers, making it difficult to use in paper form and requiring a thesaurus or coding application to find and complete ICPC-3 codes. This will represent a permanent and unavoidable barrier to ICPC-3 implementation, and will significantly impact the time required to code with ICPC-3.**
11. **Numerous classes in ICPC-3, almost exclusively new ones, do not map to either ICD10 or ICD11, mostly both, and some do not map to either ICPC-1 or ICPC-2. This is the first time that a revision of ICPC shall not map to all ICD codes. This issue arises since classes have been included which are not diseases or health problems, and do not fit the original scope of ICPC.**

# Archetypes and rubric tags

The “colour codes” historically included in the paper version of ICPC-2 (i.e. “*two-pager*” files) were useful for grouping classes according to specific aetiological attributes, such as ‘infections’ or ‘neoplasms’. However, incorporating grouping attributes in the rubric code itself would have the effect of adding another axis, or component, or structural element which would itself also have to follow the rules of non-ambiguity.

Many classes may have different aetiologies, such as pleurisy, which may be neoplastic, infectious, iatrogenic, traumatic, etc.. Assigning membership to only one group, exclusively, would be an inappropriate reduction. Such would mean that, for example, a disease can be an injury but cannot be an infection, or vice versa. It is not desirable to solve this fracture by creating different classes for different aetiologies of a class at the same hierarchical level (e.g. *neoplastic pleurisy* and *infective pleurisy*, at the same hierarchical level as *pleurisy with undefined aetiology*). ICPC prioritises localisation over aetiology, but still takes into consideration the aetiology, reflected in the numbering sequence and the colour codes. ***This double characteristic of ICPC needs to be represented properly when creating a new revision.***

An alternative proposal is to allow for different grouping attributes to be assigned to each class, and this is facilitated with an archetype model. Such grouping attributes may then be used for easy and appropriate grouping of concepts. These grouping attributes may be identified by the first letter of the group name, such as “N” for “neoplasm” or “I” for “infection.” Archetypes, distinctly from colour codes, allow each class to possess different attributes, or belong to different groups, simultaneously. Thus, the multiple aetiologies of classes, such as pleurisy, can be easily represented for grouping purposes, or to collect additional information about aetiology in an individual patient. In the case of classes with multiple attributes, the user should be encouraged to code the attribute, for additional precision. Alternatively, hierarchical expansion using mapped classifications such as ICD can help resolve additional detail on aetiology.

Other uses for archetypes and rubric tags are given below.

**Proposals:**

1. **Grouping of risk factors for retrieval, mapping, to facilitate user coding or to add individual case detail:**
   1. **Grouping of all neoplasms (possible sub-grouping into malignant, benign and other/undefined/unspecified)**
   2. **Grouping of all infections (possible sub-grouping into viral, bacterial, fungal, parasitic)**
   3. **Grouping of all injuries**
   4. **Grouping of all congenital diseases**
   5. **Grouping of process codes, reflecting the historical 2 to 6 subdivisions, which may now be collapsed**
   6. **Other groupings, similar to the original colour codes, but taking advantage of the flexibility of such tags to avoid errors which may have been committed in the past.**
2. **Identifying rubrics which should be used only in males, or females, or in defined age groups.**
3. **Labelling diagnoses which are usually only incident once (i.e. life-long), to facilitate error-trapping during coding.**
4. **Alternative views for the new AANN code structure, to allow shorthand codes similar to ICPC-2 codes (e.g. RS05 / R05S / R05 can all be linked to the concept “cough”, with “RS05” as the primary rubric code and the others as rubric tags).**
5. **Interoperability:**
   1. **Embedded links to thesauri**
   2. **Mappings to other classifications**
   3. **Other interoperability applications.**
6. **Indexing and information retrieval purposes (although this function may be more appropriate for the interface terminology which is planned to be built and linked to ICPC-3).**
7. **The archetypes and rubric tags are *not* a new axis or component, and so do not cause problems of ambiguity.**
8. **The number of components can usefully be collapsed to three: symptom label, process label and disease label, defined by rubric second alpha characters “S”, “P”(or “I” for “intervention”) and “D” respectively. The historical sub-division of process codes can be incorporated as an archetype tag.**

**Review of ICPC-3:**

1. **Chapter V section CA defines causality. Such should not be included in the core of ICPC-3, since localisation takes precedence over aetiology, and many classes in ICPC have multiple possible aetiologies.**
2. **The proposed aetiological concepts are: congenital, hereditary, infection, neoplasm, injury, lifestyle, immunology, other, unknown. It is proposed:**
   1. **That such sub-classes be removed and introduced as a separate aetiological or grouping set of attributes**
   2. **That such labels be either nouns or adjectives, exclusively, but not both**
   3. **That multiple attributes may be assigned to a class, both for more appropriate grouping, and also to allow operator coding of such attributes to better define aetiology in the single patient**
   4. **That “lifestyle” is better changed to “risk factor,” or alternatively both may be included with appropriately defined distinctions between the two classes**
   5. **That sub-divisions or more granular attributes (such as viral, bacterial and parasitic infections; benign and malignant neoplasms) be introduced.**
3. **The fact that aetiology/causality has been implemented as a set of single classes/class-kinds in Chapter V has imposed a restricted conceptualisation of aetiology as a mono-factorial concept, which is manifestly wrong. The use of such attributes should be available to the operator, but the applicability of each attribute to each main ICPC-3 class should be restricted (e.g. it should not be possible to link the attribute “infectious” to the rubric "tennis elbow”, or “congenital” to the rubric “URTI”). For grouping purposes, such links would allow the retrieval of all classes which describe, for example, neoplasm, benign neoplasms, infections, or viral infections.**
4. **The index terms are a useful feature, but some classes have a surprisingly small number of index terms, whilst others have a more extensive list.**

# Renumbering

Historically, ICPC-2 was easy to use since codes were easy to remember, with mnemonic Chapter alphas and mnemonic number sequences, with similar concepts (such as “pain” or “infections”) at similar places in the numbering sequence of each Chapter. With the new structure, a change in numbers will dis-empower users who code manually, or use three-key shortcuts in their electronic medical records. If codes are not mnemonic and the new codes are not easy to remember, then coding time will necessarily increase, since short-hand coding shall become practically impossible. In that case, new coding support tools (which are currently not available) and processes are required.

**Principles and rules:**

1. **Number (two-digit) changes should be kept to an absolute minimum.**
2. **As far as possible, the new codes should only incorporate changes in the second letter (second alpha), and not the two-digit code. Changes in the alpha and numeric components of ICPC rubrics should be minimised in the revision, to retain the skill-set of current users.**
3. **The order of concepts should not be changed arbitrarily, for the same reason.**

**Proposal:
Space may be created in all components, with minimal changes in the numbering:**

1. **The process codes may be renumbered separately from other ICPC-3 codes, or else retain the same ICPC-2 numerical digits. However, the proposal is to recode the process codes with an “*α*P” or “*α*I” prefix (“*α*” being the ICPC-3 Chapter, e.g. “*S*P01”; “SI01”), and starting with number -01. This will retain the Chapter link, as with ICPC-2, and also allow the “P01” or “I01” style abbreviation (without the Chapter alpha, equivalent to the convention in ICPC-2, e.g. “-30”) for grouping data on processes across Chapters. It is recommended to use a dash (e.g. “-P01” “-I01”) to avoid confusion with the ICPC-2 codes in Chapter P.**
   1. **By subtracting 30 units from each code number, for example (“-31” becomes “-01”), it will be easy to remember the new numbers (“A62” will become “AP32” or “AI32”, “D45” will become “DP15” or “DI15”, and so on).**
2. **The space created by freeing the numbers 30-69 may be used to extend the two-digit codes of the 1^st^ and 7^th^ component in each chapter:**
   1. **The Component 1 ragbags and “fear of” rubrics could be re-numbered to reserved numbers -41 to -49 in each chapter. Thus Component 1 shall have reserved numbers -01 to -49 (20 additional spaces, per chapter).**
   2. **The Component 7 codes may be renumbered -50 to -99, possibly with the use of the new (smaller numeric value) codes (-50 to -69) for less common disease-label rubrics, such as neoplasms of congenital malformations or even infections (19 additional spaces, per chapter). An alternative case could be made to shift all disease-label codes up by a fixed value (for example, re-start at -50) so that the numeric change is easier to remember (e.g. R74 – URTI becomes RD54 – and all other codes shift up by a fixed value of 20, like with the process codes above).**
   3. **Such would allow the creation of space to accommodate new rubrics (only 30 or so are required, all over ICPC-3), whilst allowing the old numbers to be retained for most rubrics. The numeric changes will involve less-commonly used rubrics, and most rubrics that are used frequently will retain their old numbers.**

**Review of ICPC-3:**

1. **The renumbering of ICPC-3 rubrics seems haphazard, and the mnemonic numbers of ICPC-2 are lost. In some cases almost all the classes in an entire component have been re-numbered to make space for one new rubric. For example, all Component 1 rubrics in Chapter R from “05” on were changed to add “Snoring” as RS05, when such a new code could have been inserted lower down in the numerical order. Snoring now precedes cough in the list, even though cough is the most frequent symptom presented in primary care internationally.**
2. **Historical users will thus have to re-learn all ICPC code numbers, from scratch. Some rubrics will be very difficult to remember, with multiple characters and digits.**
3. **The extensive renumbering ICPC-3 classes does not always follow the conventions and mnemonic numbering of ICPC-2. The decision to re-start the Component 7 and Process re-numbering at 01, for example, will mean that the same two-digit number refers to different classes across different Components in the same Chapter.**
4. **The numbering and subdividing of sub-sections in Chapters A1 is confusing to the new user, with many new sub-sections which do not follow standard ICPC convention.**
5. **The renumbering is inconsistent with the WICC decision to follow the “AANN” format of a single alpha Chapter code, single alpha component code, and two-number rubric code:**
   1. **with the two-character Chapter label A1, and the Roman numeral labels for Chapters I-V**
   2. **with some three-digit rubric codes (process codes)**
   3. **with the one-number-one-alpha (1N1A) Chapter II section labels**
   4. **with the extension of all Chapter II codes with an extra digit (0 to 9) to create five-character codes**
   5. **with scales included as code extensions, with their own alpha-numeric or numeric codes, which will extend the ICPC-3 codes with extra characters**
   6. **with the regional extension codes, with their additional two-digit extensions.**
6. **Consequently, and additionally, to the above, the renumbering in ICPC-3 is inconsistent with the *two-alpha-two-digit* (2A2N) numbering system decided by WICC; for example with the one-alpha-three digit (1A3N) process codes and the one-digit-one-alpha-two-digit (1N1A2N) Chapter II codes.**
7. **It shall be practically impossible to recall ICPC-3 code numbers, making it difficult to use in paper form, and requiring the use of a thesaurus or coding application to find and select ICPC-3 codes, and then apply extensions, scales and modifiers.**
8. **Coding with ICPC-3 shall be far more complicated and time-consuming than with ICPC-2. The additional information may be unreliable if the sub-divisions break the 0.5 per 1000 patient year prevalence rule, and the confidence interval of an observation becomes so wide that the observation itself becomes unusable. As such, the extra consultation time (with additional questions or processes necessary to capture the extra data for such new sub-classes and scales), and the extra coding time, will likely produce *lower quality data* since the number of observations in each of the myriad sub-divided classes will be less, and the observations thus individually less reliable. ICPC-3 in its current version is quite likely unsuitable for single-practice record organisation and data analysis due to excessive granularity.**

# Prevention

The prevention and “no-disease” codes are highly prevalent. A proposal to add granularity in this area has wide support.

This is already possible in ICPC-2 with post-coordination. If all elements of the encounter are coded, the content of the episode of care of preventive activity is more clearly defined. Thus, an episode of “A98 - Prevention” associated with a Reason for Encounter “A44 – immunization” and with an intermediate intervention “A44” clearly describes an episode of care of vaccination, which can then be further categorised with the actual vaccine given, coded with the WHO ATC system. Such rich information cannot be incorporated into a single code, especially considering the current ICPC structure and rules. Thus pre-coordinated classes (combining two or more distinct concepts) should not be the solution for the improved granularity requirement for “no disease” and prevention encounters.

Pre-coordinated classes will create issues with ambiguity, and this will be very difficult to manage. As such, the issue with prevention and the “no disease” category is best addressed by training users to code appropriately, and also by adding detailed information about appropriate post-coordination in the notes associated with the equivalent “A97” and “A98” rubrics in ICPC-3. In some countries, with limited coding systems, pre-coordinated *extensions* may offer a solution to capture billing information, for example, but these extensions should not be included in the core of ICPC.

However, the splitting of the prevention class is justified by frequency. This is best achieved by putting a class for prevention in each ICPC-3 Chapter.

**Proposal:**

1. **Creating a new chapter must follow all the ICPC rules, including having/accommodating 7 components, being appropriate for both RfE and Problem labelling, empirically supported by data from primary care, consonant with the rules of mutual exclusivity and domain completeness, and justified by frequency. The proposal for a new chapter on Personal Factors, including prevention and risk factors, is not consonant with these rules. It also deviates from the principle of using Chapter A as the default residual Chapter. It should not be implemented in ICPC-3.**
2. **To improve coding of preventive activity, suggestions for users to appropriately code requests for and actual interventions (possibly included in the notes or comments of A97 and A98) should be added to ICPC-3 prevention classes.**
3. **Having a code for prevention in each Chapter would to improve the granularity of data on prevention, especially when the RfE is not coded. *This is the preferred solution.***
4. **The idea of sub-codes (children) of the prevention rubric is also to be considered, but must respect all the structural principles and rules.**
5. **Risk factors can be coded with the archetype tags proposed above, which avoids the need for grouping in a separate Chapter. A discussion on which disease-label concepts are diseases and which are simply risk factors (e.g. diabetes type II, hypertension, hyperlipidaemia, osteoporosis, etc.) shall then be necessary.**

**Review of ICPC-3:**

1. **Prevention is now in a new Chapter, labelled A1. TGA has proposed that a class for prevention in each ICPC-3 Chapter would allow higher granularity data capture, and provide useful information about preventive activity focussed on a defined disease (e.g. vaccination to prevent hepatitis, vaccination to prevent HPV, blood test to diagnose colon cancer), without necessitating pre-coordinated classes. By moving prevention to a new Chapter, the granularity is not necessarily improved, as compared to the TGA proposal. As such this change is not desirable and has not been suggested by either the WICC Chapter Groups or the TGA.**
2. **It is unclear what prevention, family planning, general examination and “other reasons for visit” have in common to be combined in Chapter A1, and what conceptually distinguishes them from other classes which remained in Chapter A, the general Chapter.**
3. **It is also unclear what defines “other” reasons for visit from other rag-bags in Component 1 in other Chapters, especially Chapter A. It seems that the section has been created to accommodate foreign objects or organs, or an artificial stoma.**
4. **What is the definition of a “reason for visit,” how is it distinguished from a RfE?**
5. **A significant number of pre-coordinated classes have been created, combining prevention, as a purpose, linked to a defined activity or intervention:**
   1. **Classes in Chapter A1 section AG combine a process with a purpose, presumably to replace the A97 “no disease” class in ICPC-2. However, the absence of disease is not part of the inclusion criteria, and as such these classes create substantial ambiguity regarding the presence or absence of an underlying problem. The sub-divisions are also not entirely intuitive, and the expected prevalences of each class are not clear, in order to justify such sub-division**
   2. **Classes in Chapter A1 section AI combine a process with a patient treatment preference or request. This creates substantial overlap with the use of process codes to code the RfE when a patient requests a defined intervention. By combining both the discussion and the process in one class, ambiguity is necessarily created regarding whether: the patient is requesting the discussion but not necessarily the intervention; or he/she is requesting the process alone and the provider has initiated the discussion; or the provider has initiated the discussion with the patient reluctant to have the intervention, etc.. As such, the pre-coordinated term is actually much more ambiguous than the prior convention, recommending the use of process codes to code patient requests and doctor processes separately. The classes in this section also create overlap with the ICPC-2 process code -45 “advice”, which has now been removed from ICPC-3. It is not clear how the request for advice shall be coded as a RfE in ICPC-3, and whether a new special case shall be created where such a request shall now be coded with a Component 1 rubric in Chapter A1 section AI, rather than as a request for an intervention using an Intervention code.**
   3. **Classes in Chapter A1 section AP combine a process with a disease. This creates ambiguity regarding the presence or absence of the disease, especially when they are used as a problem label, since such distinction is not specified in the inclusion criteria and thus is not resolved by coding with these classes. Even if such were to be defined, and the ambiguity so resolved, these classes force the choice between coding both the process and the scope (specific disease prevention), should the disease be absent, but then only the disease should it, in fact, be present. As such, these codes do not actually add useful alternatives, when compared to standard ICPC-2 coding, but rather introduce ambiguity together with a substantial risk of not identifying cases where preventive activity, in fact, identified a disease. With the proposed system of post-coordinated classes, in ICPC-3 one can either code the process and its aim (or scope) as a combined problem label, or the actual problem itself, should it be identified. As such, less information is being captured, not more.**
   4. **Classes in Chapter A1 section AR combine the concept of the reason for visit and the presence of a device, transplanted organ or stoma, and then provide an additional rag bag class. They seem to code the actual presence of a foreign object, or opening, as the RfE, since the reason for visit (a surrogate for the RfE) concept is included in the class label. ICPC is used to code RfEs, Interventions and Diagnoses/Episode titles, and not the presence or absence of various body organs or foreign objects without such being either a RfE or a problem. The coding of such foreign objects may be added as an appendix, if required. The AR99 rag bag needs to be better defined to avoid overlap with rag-bags in other Chapters. Other AR classes also need to be better defined to avoid overlap, namely with related disease-label classes, classes AD42 “complication of medical treatment” and AD43 “side effect of prosthetic device”. Such new classes are also questionable due to low frequency argument. Finally, it must be considered that Component 1 classes are appropriately used to code both the problem label or diagnosis, and the RfE, and as such one must avoid labelling a Component 1 class as a “reason for visit.”**
6. **In Chapter I section -3, a number of new classes have been introduced which combine both a process and a problem. Such will create issues when such classes are applied across ICPC-3 Chapters, since the concept label seems to tie the process to a specific Chapter, when process or intervention codes should apply to all ICPC Chapters. The solution proposed by the Process Group in Crete, to create a process class for “formulation of a management /treatment /intervention plan,” is consistent with current ICPC convention and coding rules, and should have been adopted instead.**
7. **It is unclear why post-coordinated classes have been introduced which include both a purpose and a process, or a patient request and a process, or a process with a disease. The same aim can be achieved by separately coding both the process and the problem label, or the RfE and the process, or the RfE and the process and the problem label. However, once such post-coordinated classes were included, it is unclear why this was duplicated in both Chapter A1 and Chapter I. After all, combining processes with problem labels in one section would have sufficed. Why also then combine problems or diseases with process labels (see below), duplicating the post-coordination in two sections?**
8. **It is not clear if the concept of coding the individual elements of the encounter is to be retired, and if such elements should now be combined in post-coordinated classes in ICPC-3. Post-coordinated classes in the current ICPC-3 version, especially with respect to prevention, combine symptom (or reason for visit), process, process request and problem labels (Component 1, Component 2-6 and Component 7 classes in ICPC-2). It is not clear why the process was not extended to, for example, combine symptoms and diseases such as “cough” and “asthma” into “asthmatic cough”; or allow disease clusters such as “metabolic syndrome” or “asthmatic bronchitis” as combined terms; or even combined symptom clusters such as “nausea” and “vomiting” or “cough” and “fever”. The new standard for combining concepts is not defined. Once ICPC-3 now includes pre-coordinated terms, this change should be justified. Additionally, the reason why this process was not extended to create, in effect, a nomenclature as against a classification, should also be explained. Once the rules for a classification are not strictly applied, the argument for not including other post-coordinated terms becomes entirely arbitrary. ICPC-3 now seems to be neither a classification, nor a nomenclature, but a compromise between the two.**
9. **The Chapter of Personal Factors has been removed. This is not entirely appropriate. If ICPC-3 can accommodate different Axes, Extensions and Appendices, as we have proposed, then Personal Factors should be included as an Appendix.**
10. **Risk factors have not been grouped with an archetype attribute. This is a missed opportunity to better identify risk factors in ICPC-3, for useful grouping and analysis.**

# Clinical findings

ICPC-2 was designed to code reason/s for encounter, interventions and diagnoses (episode titles) with component 1 (symptom labels), 2 to 6 (process labels), and 7 (disease or problem labels). ICPC-2 does not cater for coding clinical findings. However, some classes in ICPC-2 have labels which represent a clinical sign (e.g. fever, high blood pressure). However, such is used to describe a symptom (e.g. fever) or a problem label (e.g. high blood pressure). These are not clinical signs *per se*.

In SNOMED-CT, ***all*** symptom label concepts found in ICPC-2 are coded as findings (observations), since symptoms are not accommodated. This is the exact opposite approach with respect to ICPC-2. For example, in SNOMED-CT the symptom “fever” is coded as “fever (finding).” To be technically correct, in SNOMED-CT you should measure the temperature and find it to be high before using the code “fever (finding)”. In order to code the symptom “fever” presented by the patient, you should double-code the “fever (finding)” with the SNOMED-CT code for “context: reason for encounter.” Such a system is incompatible with ICPC, at the fundamental level, and cannot be correctly mapped. Since SNOMED-CT does not code symptoms, mapping with other classification will resolve this major internal limitation.

If the proposal to move rubrics with the label of a clinical finding to Component 1 is followed through, the rubric for “Death”, which is clearly a clinical finding, would also have to move to Component 1. The moving of component 7 classes to component 1 on the basis of their label (specifically, referring to a clinical event) will lead to inconsistencies, and should not be pursued.

It is incorrect to state that ICPC-2 or ICPC-3 incorporates a coding system for clinical findings. Such a coding system could be, or maybe should be, developed by WICC, and this has been proposed by the Process Group. Such a future development is to be recommended, but the publication of ICPC-3 should not be delayed by such a process.

**Proposal:**

1. **Do not incorrectly refer to component 1 (symptom label) or component 7 (disease label) classes as clinical findings, since this is incorrect. ICPC codes symptoms, diseases and interventions, and not clinical findings.**
2. **Keep with the original ICPC-2 convention for rubrics labelled with a clinical finding term:**
   1. **Symptoms labelled with a clinical finding term should be in Component 1 (e.g. lump, fever, cough).**
   2. **Problems labelled with a clinical finding term should be in Component 7 (e.g. high blood pressure, abnormal enzyme levels, death).**
3. **Develop a classification for clinical findings in primary care, in future.**

**Review of ICPC-3:**

1. **Component 7 problem-label concepts which were defined by a clinical finding term in ICPC-2 (e.g. elevated blood pressure without fulfilling the criteria for hypertension; white-coat hypertension) have been conceptualised as defining an observation/clinical finding in ICPC-3, and moved to Component 1:**
   1. **Code KS51 “elevated blood pressure” is included in Component 1 of Chapter K in the current ICPC-3 version, rather than Component 7 as in ICPC-2. Its inclusion terms include “labile hypertension”; “transient hypertension” and “white coat hypertension”. Thus, such hypertension variants are considered as “symptoms, complaints and abnormal findings” in ICPC-3, whilst “primary hypertension “, class KD73 in ICPC-3, is considered a “diagnosis/diseases”. Thus hypertension is either a clinical sign or a disease, depending on whether one has taken one or more readings, or whether the aetiology is primary or “white coat” hypertension**
   2. **Classes AD96 “death”, TD66 “obesity”, TD71 “hypoglycaemia”, TD75 “lipid disorder”, could easily be similarly considered a clinical finding or an observation, and similarly moved to Component 1. They have not been so moved, and remain in Component 7. This is inconsistent.**
2. **The inclusion of clinical signs in ICPC-3 would require the systematic recording of all clinical signs, and the consequent inclusion of ICPC-3 classes for all significant states of common clinical signs or categorised body parameters, e.g. low blood pressure, high blood pressure, and normal blood pressure; low body temperature, normal body temperature, and high body temperature, etc. These classes are not available.**
3. **The shifting of ICPC-3 classes from Component 7 to Component 1 simply because they are labelled with a clinical sign or term, is inconsistent. The convention used in ICPC-2 should be retained:**
   1. **some symptom labels are defined by a clinical sign label (e.g. fever) and are included in Component 1**
   2. **some disease/problem labels are defined by a clinical sign label (e.g. high blood pressure) and are included in Component 7.**

# Standardisation of labels and accompanying text

The labels of most of the Rubric components have changed from ICPC-2 to ICPC-3. Such changes now need to be justified, and rules and definitions of each element should be provided.

Within the Task Group, consensus has formed that it is necessary to have strict rules for formatting the text labels of Rubrics.

**Proposal:**

1. **The rules for the ICPC-3 labels should be documented, discussed and implemented:**
   1. **Component 1 specifically:**
      1. **where the lay and the clinical term are synonyms, they both should be included in the rubric label with a slash between them: e.g.: haematemesis/vomiting blood;**
      2. **where the lay term is a hypernym or hyponym of the clinical term, the lay term(s) should not be in the rubric label, but should be included in the 'Inclusions' section. However, it was historically accepted that this may not always be the best solution, and that if it *must* be included in the rubric label, the two concepts should be separated by a semicolon, rather than a slash, in the rubric label;**
   2. **Both component 1 and 7:**
      1. **the rubric label should not include sub-parts of the rubric. Sub-parts (hyponyms) should be in the Inclusions, *not* in the rubric label: e.g. “*Condition* acute/chronic” from ICPC-2, should now be labelled “*Condition*” in ICPC-3 (with "acute" and "chronic" in the Inclusions). However, this does not apply should two different classes (e.g. different Rubrics for "acute" and "chronic") be appropriate.**
2. **The concepts and the actual titles of the “inclusions” and “exclusions”, “considerations”, “notes” and “ICD-10” mappings have not been carried over faithfully from ICPC-2 to ICPC-3. All changes must be justified, with appropriate references and/or documentation. It is proposed that the original ICPC-2 terms, definitions and rules have stood the test of time, and should not be changed without justification. The practice of referring to the standard classifications and definitions of diseases (ICD and DSM) should be retained.**

**Review of ICPC-3:**

1. **The suggestions above have not been incorporated in ICPC-3, with, at most, the slash being used rather than the semi-colon.**
2. **Not all classes have Inclusions, and it is not clear why many have been deleted.**
3. **Coding Criteria in ICPC-2 have now been replaced by Descriptions in ICPC-3. It is not clear why the Criteria have been removed. The coding Criteria were originally included in ICPC-2 to improve coding consistency, and not to define or describe disease entities. This would protect Wonca from being sued for taking the position of defining, or describing, a condition. The distinction is a fine one, but may be important to avoid legal implications should Wonca now become a body which defines or describes clinical entities.**
4. **Should the decision be taken to include disease label, process label or symptom label Definitions in the classification, even then the Criteria could be useful to avoid ambiguity of rubric use. As such, the Criteria need to be maintained, and updated as required in the ICPC-3 revision.**
5. **Considerations, which helped coding accuracy, have been removed from ICPC-3. This is not a positive step.**
6. **A significant number of spelling and grammatical errors have been introduced in ICPC-3, and these need to be identified and corrected.**

# Fear and functioning

The classes -26 – “fear of cancer…” and “-27 fear of other…” are consistent across chapters, and there have not been many calls for major change.

The class -28 – limited functioning/disability was frequently used in some chapters, much less so in others:

- as a RfE: A28 – 0.6/1000py; B28 – 0; D28 – 0.1; F28 – 0.3; H28 – 0.1; K28 – 0.1; L28 – 4.9; N28 – 0.6; P28 – 0.1; R28 – 0.2; S28 – 0; T28 – 0; U28 – 0.2; W28 – 0.1; X28 – 0; Y28 – 0; Z28 – 0.1 (Dutch Transition Project data, rates per 1000 patient years);
- combined RfE + Problems: A28 – 0.5/1000py; B28 – 0.2; D28 – 0.4; F28 – 1.5; H28 – 0.5; K28 – 0.8; L28 – 0.4; N28 – 0.5; P28 – 0.2; R28 – 0.1; S28 – 0; T28 – 0; U28 – 1.2; W28 – 0.1; X28 – 0.4; Y28 – 0.2; Z28 – 1.9 (ditto).

Thus, removing all -28 codes from all Chapters is not justified by the frequency data.

**Proposal:**

1. **Creating a new chapter must follow all the ICPC rules, including having/accommodating 7 components, being appropriate for RfE and Problem labelling, based on empiricism, consonant with the rule of mutual exclusivity and domain completeness, and justified by frequency. The proposal for a new Chapter on Functioning is not consonant with these rules.**
2. **A classification of functioning and disability already exists and is maintained by WHO (ICF). There is no need for WICC to create a new coding system for this purpose. The existing classification includes qualifiers for indicating severity of the disability or impairment, for the localization and the nature, etc.. Including parts of this classification in ICPC-3 is not appropriate, since concepts either do not fit the existing Chapters and their symptom, intervention, or problem components, or break the rule of mutual exclusivity due to overlap with existing ICPC rubrics. One must consider that ICF is complimentary to ICD-10, and that WHO encourages their use together (ICD-10 classifies diseases and comorbidities, ICF classifies states of health and functioning). In all utilisation cases, the health condition (disease) is coded with ICD-10, and then the functioning is coded with ICF. Thus, even in the case of ICPC-3, the use of a classification of functioning should be complimentary, and not included in the core. If ICF concepts are included in the core, their mapping to ICD-10 will be problematic.**
3. **It is proposed that all existing rubrics for “fear of” and “limited functioning” remain in Component 1 in each Chapter, as in ICPC-2. Having codes for limited functioning in each Chapter helps one understand the actual disability, not just as a functional problem, but understanding which body system is affected.**
4. **A classification of functioning and disability could be adopted (the preferred choice is ICF), modified, or created by WICC and mapped appropriately to the appropriate ICPC-3 rubrics. Such a classification could additionally address such issues as severity, temporality and other related factors. It should be considered that such attributes would also apply to related areas not directly linked to functioning, such as other symptom-label concepts in Component 1.**

According to the ICF Official manual (2013, WHO, *How to use the ICF*):

“Health conditions (i.e., diseases, disorders, injuries or related states) are classified primarily in the International Classification of Diseases (ICD) which provides an aetiological framework. The ICF and ICD are two complementary WHO reference classifications; both members of the WHO Family of International Classifications. ICF is not associated with specific health problems or diseases; it describes the associated functioning dimensions in multiple perspectives at body, person and social levels.” (Page 4)

As such ICF is not intended to be used to label a health problem or diagnosis. As such, ICF concepts have no place in the core of ICPC-3.

**Review of ICPC-3:**

1. **The removal of the -28 codes, which were present in each Chapter in ICPC-2, has removed the capacity of ICPC-3 to localise a disability, as a problem or as a RfE, to a body Chapter. This is a major regression.**
2. **The creation of a Chapter for Functional status is incongruent with the rules that each Chapter has both symptom label and disease/problem label components, that classes are included based on frequency, and that classes are mutually exclusive. See the comments in the Content section above for more detail, especially the Review of ICPC-3, points 4, 5 and 6.**
3. **The inclusion of ICF-related concepts in ICPC-3 is incompatible with the current structure of ICPC, since ICF is not designed to label a health problem or diagnosis, and certainly not the patient’s RfE.**
   1. **ICF measures functioning and health. These are very different and much broader concepts than the simple content of primary care, driven by the patient’s RfE and delivered during the consultation, centred around a problem which defines an episode of care. Functioning is related to health and illness in the community, which may be normal, or may be abnormal but not necessarily requiring health care. Thus, the combining of functioning, health, illness, disease and elements of the primary care consultation in one classification, without an appropriate conceptual framework, will simply provide placeholders for concepts which are fundamentally different, without an appropriate conceptual or categorical framework. The concepts emerge from, and describe, quite different domains**
   2. **The argument that such an inclusion will extend the domain of primary care, would require an entirely new evidence base, a new conceptual framework and new categorisations. Only then, would such new classes, as have been included in ICPC-3 Chapter II, emerge empirically. This new evidence base would have to emerge from a re-orientation of primary care away from the traditional model of the doctor-patient encounter to a more community-, or public-health-oriented, model, with entirely new methodologies and outcome measures. It would also require a fundamental re-definition of primary care to extend the models defined by Barbara Starfield, the Institute of Medicine and Wonca, to include the health problems of people who do not necessarily initiate a contact with the primary care provider. As such, it will require a re-definition of primary care from patient –centred models to health-oriented, community-based models driven by health goals, and not patient requests. Such a fundamental paradigm shift would create strong cross-over with the public health agenda and its intervention models. If this is the aim of Wonca, it requires far more than the insertion of a Chapter and 70 classes in ICPC-3. It requires a new definition of Primary Care, a new model for the consultation, new health outcome measures, and, above all, an entirely new and robust evidence base that such a shift away from the patient’s agenda to a disease-centred agenda (as in the UK with the Quality Outcomes Framework, but far more radically in ICPC-3) actually confers useful health benefits, considering the harms, and is also cost-effective. The evidence for such is, at best, controversial, and, in fact, is probably refuted by Starfield’s research which shows defined benefits in the areas of mortality, morbidity, patient satisfaction and cost with the current model based on first contact, longitudinal continuity of care, comprehensive services and coordination of health care**
   3. **Unless the definition of primary care is changed, and doctors change the way they practice primary care, the inclusion of a chapter on “health” or “functioning” in ICPC-3 is pre-mature, and not evidence-based**
   4. **However, there is no reason not to include functioning, disability and health as an Axis or Extension in ICPC-3. This would require empirical data to define the appropriate tool. Such evidence for the current Chapter II is not available.**
4. **Functional status may be normal. As such, there is a conceptual overlap between the new Chapter II classes and the ICPC-2 class A97 “no disease” and partial overlap with the ICPC-2 class A98 “prevention,” which latter classes have been re-organised in ICPC-3. As such it is difficult to understand how the concept of *“no disease”* will be coded in ICPC-3. This is a common RfE and problem label, often used when patients request certification not related to a health problem. Theoretically, with the current coding structure, the definition of “no disease” would require a code of “0” to be assigned to all 70 or so classes in Chapter II of ICPC-3. The issues with the classes in Chapter A1 section AG and ambiguity with regards to “no disease” have been discussed in the section on Prevention, above. If the classes in Chapter A1 section AG shall be used to code “no disease”, then the “0” values of the classes in Chapter II may represent a significant overlap.**
5. **Chapter II Function classes may code either a problem or the lack of that same problem, within the same class, depending on the value ascribed by the nominal/ordinal measurement (the value “0” describes the absence of a problem). This creates an anomaly which is a significant problem for meaningful analysis of functional RfEs and problems, especially in context of the fact that other ICPC-3 codes are binary or true/false (value is either *present* or *not present,* so if the code is present in an EHR cell, the condition is present) whilst the Chapter II codes have nominal or ordinal values, so that the *presence* of the code may mean that the condition is *not present*, should the value be zero.**
6. **It is actually unclear whether Chapter II classes will have a nominal or ordinal value, or both, or an optional true/false mode, with an option to choose a sub-scale. If they are not binary, this will create another inconsistency in the interpretation of data across other Chapters with simple binary (present/not present) classes. It is also unclear how the nominal or ordinal values will be determined differentially as a RfE and an episode title.**
7. **It is unclear whether the new ICPC-3 Chapter II class nominal codes (0,1,2,3,4,9) can be interpreted as ordinal (especially considering the -9 value being “not applicable”), and how such values should be analysed, especially in conjunction with other ICPC-3 classes which do not have default ordinal scales. It is also unclear what the meaning of such ordinal scales is, and the intervals between these numbers, especially when applied across so many ICPC-3 classes across the domain of functioning and health. Could such ordinal data be combined, between Chapter II classes, or with other ICPC-3 data, and how? What would be the meaning of such combined data, and especially the combined values? The academic base for such ordinal scales being included in ICPC-3 should be defined and shared, and especially on how such scales will affect the validity and reliability of ICPC-3 data.**
8. **Many rubrics in Chapter II overlap with symptom label and problem label codes, and even interventions (measurement of bodily functions which define a functional status) in other Chapters or sections of ICPC-3. Examples include codes for social and family relationships, social, neurological, hearing, locomotor, psychological, and vision problems, pain and drug adverse effects. This represents a significant fracture in the mutual exclusivity of classes in ICPC-3.**
9. **ICD and ICF are separate classifications, and the inclusion of ICF in ICPC-3 will create an irremediable inconsistency with ICD-10 and ICD-11. In fact, many Chapter II classes do not map well to other major classifications or coding systems, including ICPC and ICD. This will create major issues with ICPC-3 users who wish to output in ICD-10 or ICD-11.**
10. **The definition of functioning as a concept within ICPC-3, the actual Chapter II description, and the class selection process (from the available ICF classes) are not well defined, and the academic background is unclear. The Description for Chapter II defines its purpose as the measurement of function and functioning-related aspects for patients in contact with primary and community care, which is very broad. However, the classes included in Chapter II are a selected sub-set of ICF. As such, domain completeness is not guaranteed, due to this reduction. Many aspects of functioning have been included in ICPC-3, but have all possible aspects been included? Is spirituality, for example, or love, relevant to health status? ICPC-3 has mostly been developed from hard data from international primary care, collected over decades with ICPC-2. How can a sub-set of ICF be included in ICPC-3 without such an international database?**
11. **The inclusion of a subset of ICF within ICPC-3 (as against a mapping to code numbers, such as is the case with ICD-10 and Snomed) will require that a licence for the use of ICF will be, in turn, required to use ICPC-3**

**Recommendation:**

**Functional status has been associated with ICPC, as a separate assessment modality, since its inception. It should be incorporated as a separate Axis or Extension, using -28 classes in each Chapter as a placeholder. Empirical data is required to develop a reliable and valid tool.**

# Joining chapters X and Y, and making Chapter W unisex

Chapters X and Y will be joined as Chapter G, to remove the sex-linked codes in ICPC-3. The justification for this decision was that information regarding patient sex is available from the medical record, and thus does not need to be captured by the classification. However, the reason for separating rubrics in the sex-linked Chapters in ICPC-2 was more to do with the actual nature of sex-linked diseases and symptoms, which are innately linked to the patient’s sex.

Thus, despite the proposed change, many rubrics in the proposed new Chapter G will still be innately sex-linked (such as “prostate cancer,” “penis pain,” “vulval pruritus” and “ovarian cyst”). As such, the join does not resolve the sex linkage issue. Rubrics will not be unisex. Similar issues will arise in Chapter W, and in fact may be much more problematic since most concepts are inherently sex-linked.

This creates a major fracture, with many sex-linked (male or female) symptoms but more unisex disease concepts. The analysis of diagnostic associations will require post-hoc sex identification of male and female patients labelled with the disease, since the classification alone will not be able to resolve this. The analysis of the diagnostic association between “breast lump –female”, and “breast cancer – female”, will be impossible in ICPC-3 unless the data has a separate tag for male and female patients, or is linked to other data from electronic health records which allow determination of the subject’s sex. The diagnostic association between a symptom and a genital disease may be quite different for the two sexes, and it is essential to retain the capability to make such an analysis. In situations where such information is not reliably available, ICPC-3 cannot be used to study such diagnostic associations, or to reliably measure the prevalence of sex-linked diseases.

Once you lump you cannot split. However, we propose a better way to reduce the sex-linkage of genital diseases which are not substantially different in the presentation in different sexes

**Proposal:**

1. **One position supported in the Task Group is to keep the original ICPC-2 Chapters W, X and Y. It would be possible to only join those rubrics in Chapter X and Y which are genuinely unisex.... such as “syphilis,” “gonorrhoea,” and “herpes.” These could be moved to Chapter A or Chapter U, even though they fit better in Chapter X/Y/G. However, this proposal has been previously rejected by the full WICC Committee.**
2. **An alternative solution, supported by the Task Group, is to merge Chapters X and Y to create a Chapter G with three alphas available for each rubric (“G” or “X” or “Y”) depending on the following rules:**
   1. **“X” for those rubrics which are exclusively female;**
   2. **“Y” for those rubrics which are exclusive to males;**
   3. **“G” for unisex rubrics.**
3. **With this proposal, it is also possible for the user to be offered the choice of using different letters in different contexts:**
   1. **“G” being most appropriate in datasets with available patient sex identifier data;**
   2. **“X” and “Y” being most appropriate when sex identifier data is not available, and so must be captured using the Classification.**
   3. **For this to work, such “joined” codes will need to have the exact same two-digit code, so that the rubric meaning would not be changed by assigning “X”, “Y” or “G”. This is the same as using process codes without a chapter identifier (e.g. -31) for grouping purposes, with the “G” being the grouping of the two sexes just as the “-“ allowed grouping of process codes across chapters .**
4. **Chapter W should remain sex-linked, since the concepts within are innately linked to the female sex.**

**Review of ICPC-3:**

1. **Chapter W has been split, and family planning included in Chapter A1. However, many classes which moved from Chapter W to Chapter A1 deal with the control of fertility, and many classes which have remained in Chapter W deal with pregnancy and the puerperium. As such, there is more commonality between the classes which have moved from Chapter W to A1, than there is between those same classes and the prevention and other reasons for visit classes which populate the rest of Chapter A1. As such, the move of classes from Chapter W to Chapter A1 seems to create a mismatch. This move was not suggested by either the Chapter Group or the TGA, and its justification is not provided. It should be reversed.**
2. **Chapter G has been finalised. All concepts have the unisex Chapter alpha “G.” However, many classes in Chapter G still have sex-linked class labels:**
   1. **Chapter G classes include such inherently male sex-linked concepts as GS01 “pain penis”; GS02 “pain testis/scrotum”; GS20 “penis symptom/complaint”; GS21 “scrotum/testis symptom/complaint”; GS23 “prostate symptom/complaint”; GD10 “prostatitis/ seminal vesiculitis”; GD11 “orchitis/epididymitis”, etc.**
   2. **Chapter G classes include such inherently female sex-linked concepts as GS05 “menstrual pain”; GS06 “intermenstrual pain”; GS07 “menstruation absent/scanty”; GS08 “menstruation excessive”; GS09 “menstruation irregular/frequent”; GD09 “pelvic inflammatory disease”; GD25 “malignant neoplasm cervix”; GD29 “fibromyoma cervix/uterus”; GD65 “cervical disease”, etc.**
   3. **The split of Chapters X and Y in ICPC-2 allowed easy error-trapping with classes which were inapplicable in females in Chapter Y, and classes which were inapplicable in males in Chapter X. This can now be handled with an archetype rubric tag for male-only and female-only classes, which should be added in ICPC-3.**
   4. **The split of sex-linked codes in ICPC-2 allowed better discrimination of diagnostic associations between sex-linked RfEs and sex-linked genital disease. For example a breast lump in a female and its association with female breast cancer may exhibit entirely different diagnostic associations from a male breast lump and male breast cancer. Such a distinction will have to be made outside of the classification for the first time, with data on patient sex not captured in the classification, but sourced from other fields in a database. This limitation may not have been adequately considered in implementing this join of Chapters X and Y into G.**
   5. **The proposal from the Chapter Group review and the TGA was to merge Chapters X and Y into G, as decided by WICC, but to maintain the X and Y rubric alphas for those classes which were innately sex-linked. It was proposed to use the G rubric alpha for those classes which were not substantially different in males and females, such as sexually transmitted infections. A deeper review could identify other conditions where the manifestation of a disease is very different in males and females, such as breast cancer, and possibly extend this proposal to such conditions. This would allow a Chapter G revision which joins genital diseases into one chapter but retains a sex-linked Chapter alpha when appropriate, but assigns a neutral “G” Chapter alpha for those that are not.**

# Personal and risk factors

The issue of personal factors emerged from small group discussions in WICC. Factors such as age, sex, gender, height, ethnicity, social status, hobbies, habits, religion, occupation, etc. are not captured by ICPC-2. Such is correct and consistent, since such factors are not part of the domain of primary care, and especially not part of the reason for encounter, intervention and diagnosis model of the encounter. As such, they are not part of the core of ICPC-2, and should not be included in the core structure of ICPC-3 without a change in the fundamental rules of ICPC. However, such a formal rule has never been proposed to, or adopted by, WICC. In fact, the discussions have always leaned towards a separate coding system for personal factors

Personal factors should be, and often actually are, recorded in other parts of the medical record, and separately retrieved for analysis as appropriate. When such personal factors actually are an RfE or a problem, then they should be double-coded with both ICPC-3 codes and personal factor codes (e.g. age and age-related dementia; financial problems (as an RfE) and poor socio-economic status). This is equivalent to the current situation in ICPC-2, when personal factors which were presented as a RfE or became a diagnosed problem, were coded with ICPC-2, but other personal factors were not.

It is a procedural fracture to exclude sex (a personal factor) from ICPC-3 by joining “X” and “Y” into Chapter “G” but then introduce personal factors as part of the Social Chapter “Z”.

The move of certain rubrics from the General Chapter “A” to another location inn ICPC-3 is also inconsistent with the rule that Chapter “A” is the residual chapter for those rubrics which are not linked to a body system.

The grouping of risk factors in ICPC-3 will be easier should the archetype proposal be implemented. As such, the need to move risk factors to one Chapter will be eliminated. It is desirable to have risk factors in appropriate Chapters, as in ICPC-2, but now also to have the facility to label a condition (either a symptom or a disease) as a risk factor, using the archetype model.

**Proposal:**

1. **Personal Information and Personal Factors are not to be included in the core structure of ICPC-3. They should not be coded with ICPC-3 codes unless they are presented as a RfE or diagnosed as a Problem. This is already the case with Chapter Z in ICPC-2, and this approach should be retained.**
2. **A classification of personal factors needs to be adopted, modified or created by WICC and mapped to appropriate ICPC-3 rubrics. This coding system should capture data on factors influencing health including social, environmental and personal factors, occupation, hobbies and habits, health approach, and possibly attitudes, religion, spirituality and belief.**
3. **All risk factors should be grouped using archetype tags. It is not useful to remove risk factors from each of the main ICPC Chapters, simply in order to group them.**

**Review ICPC-3:**

1. **Personal Factors have been removed from Chapter ZC. Such Personal Factor classes may be introduced as a new Appendix in ICPC-3.**
2. **Risk factors could be identified with an archetype tag which would allow useful grouping for analysis, and also retrieval, purposes.**

# Process

The Process Group met in September 2019, before the Crete WICC meeting, and made proposals for process codes in ICPC-3. A review of the actual implemented changes was performed in April 2020 and is currently under review by Task Group A for eventual feedback to the Consortium.

**Review of ICPC-3:**

1. **Process codes have now been moved to a new Chapter I, which includes processes and interventions. The new process codes have three digits, and the new format is different from other ICPC-3 codes. This may be a barrier to the goal to have mnemonic codes in ICPC-3.**
2. **Some process codes, especially in section I3, have a defined purpose included in their concept label, and as such are not compatible with all Chapters. Examples include -301 “cardiovascular program”; -302 “heart failure program”; -303 “dementia (management) program”, etc. Consequently, such codes are not applicable for most ICPC-3 Chapters, which is another departure from the standard ICPC convention.**
3. **Process Group proposals formally agreed in Crete in September 2019 have not been implemented, and other decisions made in their place. Examples include:**
   1. **All process codes should be applicable in every Chapter of ICPC-3**
   2. **Component 3 becomes "Medication, vaccination, treatment, therapeutic interventions"**
   3. **Component 6 becomes "Coordination, referral and other reasons for encounter"**
   4. **Proposals for a split of -41, and component changes concerning classes -46 and -47**
   5. **A new class for management plans, in contrast to the section with classes including a defined purpose which has been currently implemented in ICPC-3.**
4. **New classes have been included, and others retired, independently of the recommendations from the Process Group:**
   1. **A class has been created for quaternary prevention. The prevention of harm from an intervention, including preventive activity, is not a separate activity, but is part of the clinician’s thought process as an intervention is considered. As such, it should not be a separate class, but an attribute or characteristic of the preventive activity itself, which is appropriately coded with another class outside the process codes. As such, the inclusion of the -213 “quaternary prevention” class separately from primary, secondary and tertiary prevention, and as an intervention class, represents an anomaly. Preventive activity should be coded with one class, in each Chapter of ICPC-3, and further sub-divisions may be handled by an extension or by double-coding “no disease,” or the relevant disease, as appropriate**
   2. **The process code -45 “Observation/health education/advice/diet”, has now been removed from ICPC-3. It is not clear how a patient’s request for advice, etc., shall be coded as a RfE in ICPC-3. This was one of the most frequent requests for an intervention coded as a RfE, and in fact it was the third most common such RfE. It is thus not clear whether an intervention class shall be used to code the RfE, as a request for that intervention by the patient, in all cases, including a request for advice. Currently it seems that an exceptional case now exists in ICPC-3 where a request for advice can now only be coded with a pre-coordinated Component 1 rubric from Chapter A1 section AI. It is not clear what defines the special circumstances for such an exception, and why this is not the case for other requests for interventions.**
5. **The Process group has made new recommendations after reviewing the changes implemented in the ICPC-3 browser, and these are currently under review by the TGA. On completion of this review, a new file shall be sent to the Consortium.**

# Review of code changes implemented in ICPC-3

Up till the September 2019 WICC meeting in Crete, the following changes were recommended by the TGA:

1. Many changes to inclusions, exclusions, considerations, and notes.
2. Major rubric changes:
   1. 4 rubrics to change chapter, 4 to consider changing chapter;
   2. 8 code deletions, with 2 more to consider;
   3. 24 new codes, possibly another 7 if frequency data supports this;
   4. 5 codes to be merged into another, with 1 more to consider.

**Proposal:**

**All changes actually made to ICPC-3 by the Consortium working group should be reviewed by the TGA. Since there is, unfortunately, no version control for the Consortium work process, this will require a separate fixed dataset to be created. Such a version would allow review of all rubrics by the TGA, without changes being made independently by other users of the ICPC-3 browser.**

**Review ICPC-3:**

1. **A formal review of all the changes implemented in moving from ICPC-2 to ICPC-3, code by code, requires time. However, it is clear that a number of changes have been made which deviate significantly from the recommendations of the original Chapter Group within WICC, the review of the TGA and/or a formal decision during a WICC meeting, or at least ignore the recommendation to return a counter-proposal to the TGA or WICC for discussion. Some examples illustrate how even minor changes may be important and should be reviewed:**
   1. **“Rhinosinusitis” has been included as a class in ICPC-3, and “sinusitis” has been removed, even though the class “sinusitis” exists both in ICD-10 and ICD-11. This should have been further discussed between TGA and the Consortium working group, but it was not. Sinusitis, as a distinct clinical concept, has disappeared from ICPC-3. Both rhinosinusitis and sinusitis may be included in the short title for the class, as a compromise**
   2. **“Sleep-related breathing problems” is the term for the concept “sleep apnoea”, which is also the way it is labelled in ICD-10 and ICD-11. This is now included in Component 1 of Chapter R, when the recommendation from the TGA was to include a new code for sleep apnoea, as a disease or problem label in Component 7.**

# Solutions

The purpose of this report is not to hamper the progress of ICPC-3 development. Rather, it is intended to re-align such to scientific norms and published international standard presentations. As such, solutions are being proposed to the issues identified in this review.

**Proposed solutions:**

1. **Governance issues should be resolved to harmonise workflow.**
2. **Authorship issues should be resolved, appropriately assigning intellectual property and formally recognising it.**
3. **The editorial control of ICPC-3 should be assigned to the full WICC Committee, and delegated appropriately.**
4. **Membership of ICPC-3 task groups should be open to all WICC members, based on sound academic background and technical expertise.**
5. **A robust structure for ICPC-3 should be defined:**
   1. **Including Functioning as an Axis or Extension, linked to the core ICPC-3 with the code for “disability” (RfE or problem) as a placeholder or link**
   2. **Creating Axes or Extensions for drugs, laboratory and imaging tests, and clinical findings with the appropriate placeholders or links**
   3. **Creating Appendices for personal factors and severity indices**
   4. **Clarifying rules for use of indices/scales, extensions and function classes**
   5. **Adopting, or modifying, existing coding systems for the above purposes, or creating new ones as appropriate, as an ongoing process**
   6. **Documenting all changes in a continuous log file.**
6. **Revising ICPC-3 classes should now involve:**
   1. **Re-numbering in a mnemonic way**
   2. **Re-organising Chapter G with due consideration of sex-linked and non-sex-linked rubrics**
   3. **Re-assigning family planning and prevention to their appropriate Chapters**
      1. **Family planning to “W”**
      2. **Prevention with a class in each Chapter**
   4. **Changing the Process codes Alpha and numbers, and revising classes following the Process Group recommendations**
   5. **Revising all other rubrics for consistency with defined principles and rules, especially mutual exclusivity**
   6. **Reviewing rubric labels for consistent formatting, and addressing any spelling or grammatical errors.**
7. **Submitting the finished product to external review by a board of experts outside WICC, and then to WICC itself.**

# Summary

The revision of ICPC-3 has progressed significantly since the creation of the ICPC-3 Consortium. However, the current governance issues present challenges to the authorship role traditionally held by WICC, and the editorial role designated to the Task Group Alpha (TGA) within WICC. The authorship role of individual WICC members is also unclear, with discontinuity between past and current WICC and TGA decisions, and other decisions taken by the Consortium.

The progression of the revision, as defined and executed by WICC, involved extensive Chapter Group and Process Group work, harmonised by the TGA. This work was guided by international datasets from primary care and family medicine, and followed established ICPC principles and rules extensively published as an international standard presentation. This work is largely complete, now, and has been passed on to the Consortium. The Consortium, instead, extended its tasks and roles, made decisions which, in many cases, were inconsistent with decisions taken on WICC, and did not formally solicit or follow further guidance from or reviews by the WICC TGA.

Currently, the version of ICPC-3 presented in the Consortium browser tool:

1. Lacks a formal foundation layer and published empirical database
2. Extends the domain of primary care without a formally published academic base
3. Includes functioning, health and prevention (both activity and purpose) in its core, but excludes clinical signs, drugs and personal factors
4. Lacks formally defined principles and rules, or is inconsistent with established ICPC principles and rules
5. Exhibits inconsistent hierarchical structure, categorisation of Chapters and class categorisation and content
6. Does not fully implement mutual exclusivity
7. Takes an inconsistent approach to pre- and post-coordination
8. Has heterogeneous granularity
9. Has both nominal and ordinal codes
10. Has an inconsistent rubric numbering system, which is difficult to memorise
11. Does not implement archetype tags, which would allow better handling of class attributes
12. Risks poor validity and reliability with new measures and scales, including undefined measures of functioning
13. Includes regional extensions in an international core classification
14. Includes grammatical and spelling errors, with rubric labels not being formally standardised
15. Involves significantly increased burdens on coders, with a complicated coding system
16. Risks copyright, intellectual property and licensing issues with the publishers of ICF
17. Exposes Wonca to potential legal issues with publishing disease definitions, rather than simply criteria for coding.

Such issues could be easily resolved, at least conceptually, with the creation of new Axes, Appendices and Extensions, and the adoption and application of a formal set of principles and rules. A robust example of such principles and rules has been developed by WICC and its Task Group, which would allow consistency with historical ICPC principles and rules, and elegantly accommodate all the new ideas accepted by the Consortium partners.

It is proposed that a formal review of ICPC-3 is needed, along the lines of the original plan approved by WICC. The original ICPC review implemented by WICC, through the Chapter and Process groups, resulted in timely and consistent proposals for change which did not introduce internal inconsistencies, especially after harmonisation by the TGA.

Such a revised ICPC-3 could be published in Alpha form in the near future, allowing for continued improvement. This would allow the eventual publication of a Beta and Final release with robust internal consistency and a coherent conceptual model, and with new characteristics which would address all the desired changes proposed by the Consortium partners.
